# Hypoxia inducible factor‐2α importance for migration, proliferation, and self‐renewal of trunk neural crest cells

**DOI:** 10.1002/dvdy.253

**Published:** 2020-09-26

**Authors:** Camilla U. Niklasson, Elina Fredlund, Emanuela Monni, Jessica M. Lindvall, Zaal Kokaia, Emma U. Hammarlund, Marianne E. Bronner, Sofie Mohlin

**Affiliations:** ^1^ Translational Cancer Research, Lund University Cancer Center at Medicon Village, Lund University Lund Sweden; ^2^ Division of Pediatrics, Department of Clinical Sciences Lund University Lund Sweden; ^3^ Laboratory of Stem Cells and Restorative Neurology, University Hospital Lund Sweden; ^4^ Lund Stem Cell Center Lund University Lund Sweden; ^5^ National Bioinformatics Infrastructure Sweden (NBIS), Science for Life Laboratory, Department of Biochemistry and Biophysics Stockholm University Stockholm Sweden; ^6^ Division of Biology and Biological Engineering California Institute of Technology Pasadena California USA

**Keywords:** embryogenesis, HIF‐2α, migration, neural crest, stem cells, trunk neural crest

## Abstract

**Background:**

The neural crest is a transient embryonic stem cell population. Hypoxia inducible factor (HIF)‐2α is associated with neural crest stem cell appearance and aggressiveness in tumors. However, little is known about its role in normal neural crest development.

**Results:**

Here, we show that HIF‐2α is expressed in trunk neural crest cells of human, murine, and avian embryos. Knockdown as well as overexpression of HIF‐2α in vivo causes developmental delays, induces proliferation, and self‐renewal capacity of neural crest cells while decreasing the proportion of neural crest cells that migrate ventrally to sympathoadrenal sites. Reflecting the in vivo phenotype, transcriptome changes after loss of HIF‐2α reveal enrichment of genes associated with cancer, invasion, epithelial‐to‐mesenchymal transition, and growth arrest.

**Conclusions:**

Taken together, these results suggest that expression levels of HIF‐2α must be strictly controlled during normal trunk neural crest development and that dysregulated levels affects several important features connected to stemness, migration, and development.

## Key Findings

- HIF‐2α is expressed in trunk neural crest cells in human, mouse and avian embryos
- Up‐ or downregulation of HIF‐2α affects trunk neural crest cell migration and proliferation
- Knockout of HIF‐2α enhances trunk neural crest cell self‐renewal
- RNA sequencing data reflects the in vivo phenotype

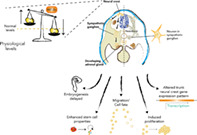

## 1 INTRODUCTION

The neural crest is a multipotent stem cell population that is unique to vertebrate embryos. Originating from the ectodermal germ layer, premigratory neural crest cells arise in the dorsal neural tube during neurulation and are characterized by expression of transcription factors like *FOXD3*, *TFAP2*, and *SOXE*.^1^ Neural crest cells subsequently undergo an epithelial‐to‐mesenchymal transition (EMT) to delaminate from the neuroepithelium, then migrate extensively throughout the embryo, populating distant sites. Upon reaching their final destinations, neural crest cells form a large variety of cell types, as diverse as elements of the craniofacial skeleton, melanocytes of the skin, adrenal chromaffin cells, and sympathetic neurons and glia.^2^, ^3^, ^4^, ^5^

Under normal conditions, hypoxia inducible factor (HIF)‐2α is stabilized at low oxygen levels and responds to hypoxia by initiating a transcriptional program for cellular adaptation to changes in energy demand. Tumor cells that express high levels of HIF‐2α together with numerous neural crest markers have been detected in perivascular niches despite the access to oxygen in these areas.^6^, ^7^, ^8^ Accordingly, HIF‐2α can become abnormally stabilized at physiological oxygen tensions (~5% O_2_) in vitro.^6^, ^9^

Previous studies in chick, quail and Xenopus embryos have shown that related *HIF1A* (encoding HIF‐1α) and *ARNT* (encoding HIF‐1β, transcriptional binding partner of both HIF‐α isoforms) genes co‐localize and are ubiquitously expressed within the developing embryo, as investigated at time points up to HH14 (HH stages in chick embryos).^10^, ^11^, ^12^, ^13^, ^14^ *EPAS1* (encoding HIF‐2α) is however expressed in a more distinct pattern and in tissues not expressing *HIF1A* (extraembryonic and endothelial cells).^12^ Embryos experience a milieu with low oxygenation (~5% O_2_), particularly before the blood circulation is fully functional, which starts at stage HH14.^11^ Despite this, HIF‐2α is not ubiquitously expressed. In addition, trunk neural crest cells form mainly after commencement of vasculogenesis and hence are not affected by high (20%‐40%) oxygen.^11^ This is in concordance with data from Barriga et al, suggesting that HIF‐α stability in neural crest cells can be controlled by both oxygen‐dependent as well as oxygen‐independent mechanisms^14^ as suggested in other systems, including neuroblastoma.^9^

Here, we explore the role of HIF‐2α during normal development up to the time point when trunk neural crest cells have completed emigration and begin to populate sympathetic ganglia. We show that HIF‐2α is expressed in migrating trunk neural crest and sympathetic neuroblasts in human, murine, and avian embryos. RNA sequencing of trunk neural crest cells with dysregulated HIF‐2α levels demonstrates a shift in the global transcriptional program, resulting in enrichment of genes associated with tumor morphology, invasion, EMT, and arrested embryo growth. Knockdown and overexpression experiments in chick embryos in vivo result in a delay in embryonic growth, altered expression of trunk neural crest genes, increased proliferation and disrupted trunk neural crest cell migration. Consistent with this, in vitro HIF‐2α knockout crestospheres display increased self‐renewal capacity. The results suggest that expression levels of HIF‐2α must be strictly controlled for proper neural crest development. These findings enhance our understanding of how genes dysregulated in normal development and tumor cells connect, and how oxygen sensing HIF‐2α plays noncanonical roles during trunk neural crest development.

## 2 RESULTS

### 2.1 HIF‐2α is expressed in migratory trunk neural crest cells in chick embryos

**FIGURE 1:**
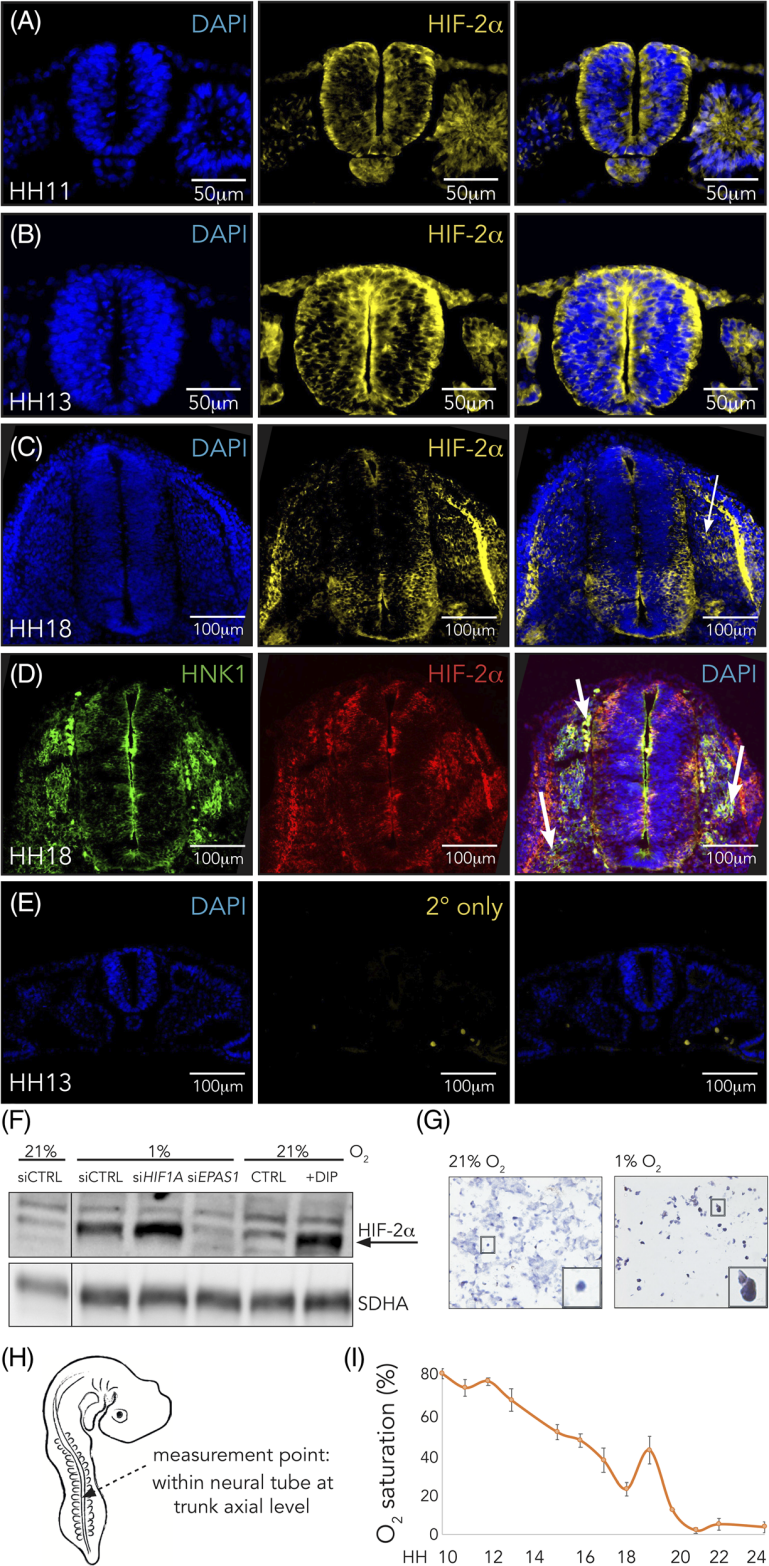
Hypoxia inducible factor (HIF)‐2α is expressed in trunk neural crest cells. A,C, Immunostaining of HIF‐2α in sections from trunk axial level of wild‐type chick embryos at HH11, A, HH13, B, and HH18, C. Arrow denotes ventrally migrating HIF‐2α positive cells. D, Co‐immunostaining of HIF‐2α and HNK1 (marker of migrating neural crest) in sections from trunk axial level of wild‐type HH18 chick embryos. Arrows denote migrating cells double positive for the two proteins. E, Sections of HH13 wild‐type embryo immunostained with DAPI for visualization of nuclei and secondary antibody only (donkey anti‐rabbit Alexa Fluor‐546). F, Western blot analysis for detection of HIF‐2α protein at 21% and 1% O_2_ following siRNA mediated knockdown of HIF‐1α or HIF‐2α. DIP treated cells were used as a positive control and SDHA as loading control. Lanes between 21% and 1% siCTRL were removed from this figure, indicated by the black line. G, Immunohistochemical staining for HIF‐2α in sections of SK‐N‐BE(2)c neuroblastoma cells cultured at 21% or 1% O_2_. H, Schematic of where oxygen measurements were performed. I, Oxygen saturation in the trunk of chick embryos during development measured ex ovo using microsensor technique. Error bars represent SEM, n ≥ 3 biologically independent replicates for each time point

The presence of neuroblastoma cells expressing HIF‐2α in perivascular tumor niches indicates poor prognosis. That these cells express stem cell‐ and neural crest associated proteins raises the intriguing possibility that they may constitute a tumor‐initiating subpopulation resembling embryonic neural crest cells. As a first step in exploring the role of HIF‐2α in the embryo, we examined its spatiotemporal expression during normal trunk neural crest development. To this end, we performed immunocytochemistry in transverse sections through the trunk axial level of stage HH11, HH13, and HH18 embryos. We detected low levels of HIF‐2α protein in neural crest cells within the neural tube of HH11 and HH13 embryos (Figure 1A,B, respectively), as well as other sites in the embryo. This contrasts with previous reports on HIF‐2α reporting expression exclusively in extraembryonic tissue at these stages.^13^ Indeed, we also detect HIF‐2α staining in extraembryonic tissue of HH11 and HH13 embryos (Figure 1A,B). Differences in results may be due to different detection methods (eg, in situ hybridization in previous reports vs antibody staining here), staging, or species differences. At these stages, trunk neural crest cells are still premigratory, and although not all cells within the neural tube will emigrate, a large fraction of these cells will generate progeny that become *bona fide* neural crest cells. We further detected HIF‐2α in cells that had delaminated from the neural tube and initiated migration in older embryos (HH18; Figure 1C), in line with data from Nanka et al.^12^ To identify these cells as trunk neural crest cells, we co‐stained with HNK‐1 antibody (Figure 1D). Secondary antibody alone confirmed that there was no nonspecific binding (Figure 1E), and we ruled out that the primary antibody (ab199, rabbit anti‐HIF‐2α; Abcam) also detected related protein HIF‐1α by knocking down both HIF‐α isoforms and blotting for HIF‐2α. The antibody did not detect any protein in the HIF‐2α siRNA lane, ensuring specificity (Figure 1F). Along the same line, we used immunohistochemistry to stain cells cultured at normoxia (21%) or hypoxia (1% O_2_) with the NB100‐132 primary antibody (mouse anti‐HIF‐2α; Novus Biologicals) and as expected only observed HIF‐2α expression at lowered oxygen concentrations (Figure 1G). Together with previous data on these antibodies,^6^, ^9^, ^15^, ^16^ these results ensure antibody specificity.

### 2.2 Development from environmental to physiological oxygen

In adult vertebrate animals, HIF‐2α is canonically induced at low oxygen levels. To understand variations in oxygen consumption during the developmental stages of interest, we measured O_2_ saturation in real time in the developing chick embryo utilizing STOX microsensors. Oxygen availability is referred to as changes of full saturation, meaning that anything below 100% saturation reflects a reduction from what liquid would hold if in equilibrium with air, which is to be expected when organisms develop into 3D structures. Embryos were removed from the egg at desired developmental time points (minimum three embryos per time point) and oxygen saturation was measured specifically within the neural tube at the trunk axial level (Figure 1H). The handling of embryos outside the egg did not change intratissue oxygen saturation over the first 4 hours. Since our measurements were performed within 30 minutes, we believe that these numbers reflect near‐endogenous levels. Within the trunk neural tube, oxygen saturation starts out high (up to 85% ± 5 SEM O_2_ saturation) at trunk specific premigratory to migratory stages of neural crest development (HH10‐HH16) and gradually decreases (Figure 1I). At the time when the majority of trunk neural crest cells have delaminated from the tube (HH18), oxygen saturation is low (23% ± 10 SEM O_2_ saturation), only to rise and fall again at later time points (Figure 1I).

### 2.3 HIF‐2α is expressed in sympathetic neuroblasts in human and mouse embryos

*EPAS1* knockout mice have severe abnormalities in the sympathetic nervous system (SNS)^17^; consistent with this, there is some, albeit limited, data suggesting that HIF‐2α is expressed in sympathetic chain ganglia up to murine day E11.5 (corresponding to human embryonic week 5). Moreover, mice lacking *PHD3* (HIF prolyl hydroxylase), a gene critical for regulation of HIF‐2α, display reduced SNS function that is rescued by crossing these mutants with EPAS1^+/−^ mice.^18^

**FIGURE 2:**
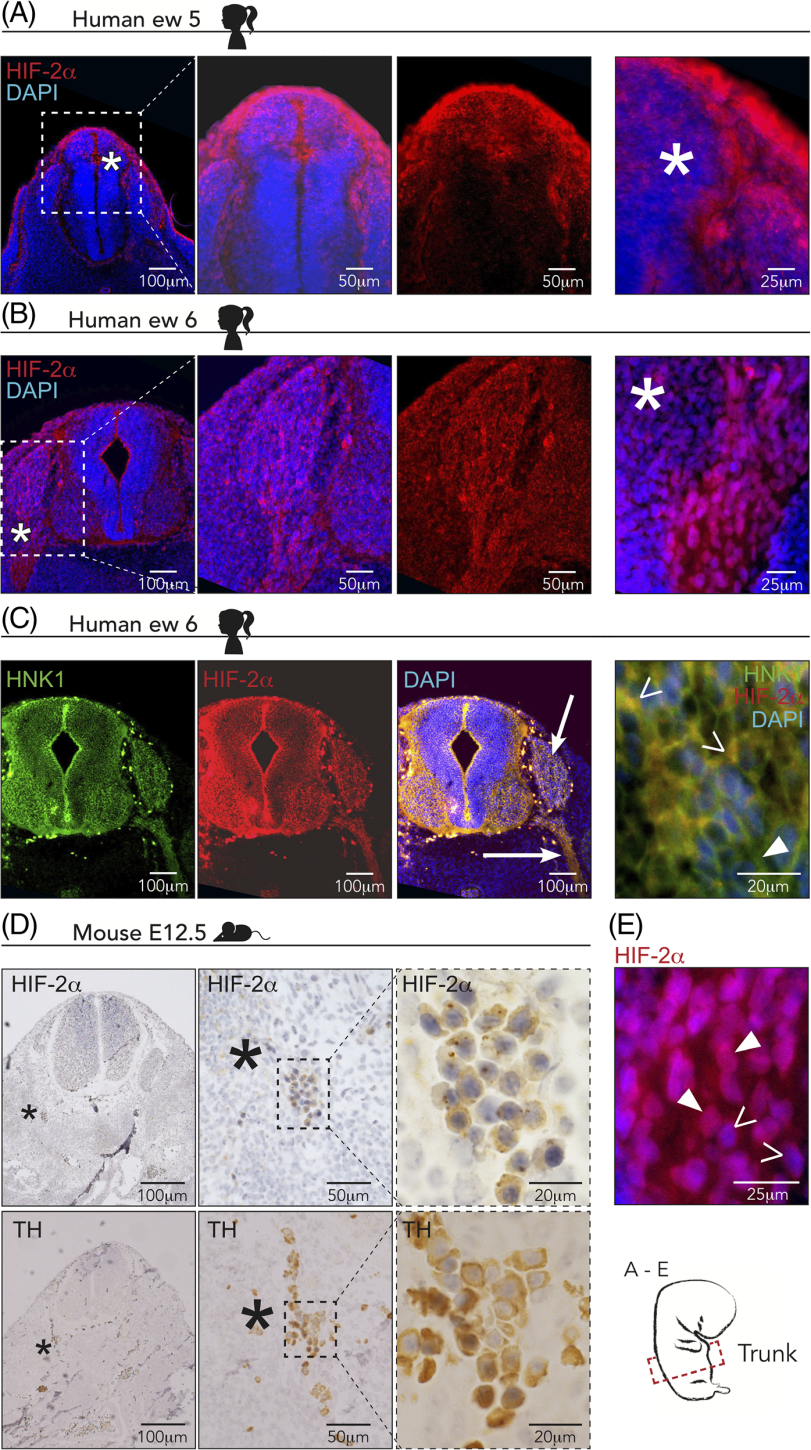
Hypoxia inducible factor (HIF)‐2α is expressed in human and mouse trunk neural crest cells. A,B, Immunostaining of HIF‐2α in sections from trunk axial level of human embryos at embryonic week 5, A, and embryonic week 6, B. Asterisks denote magnified area in the two right panels. ew, embryonic week. DAPI was used to counterstain nuclei. C, Co‐immunostaining of HIF‐2α and HNK1 (marker of migrating neural crest) in sections from trunk axial level of human embryos at embryonic week 6. Arrows denote areas staining positive for both proteins. Right panel: open arrowheads denote double positive individual cells; closed arrowheads denote cells positive for HNK1 alone. D, Immunohistochemical staining of HIF‐2α and TH in adjacent sections from a mouse embryo at embryonic day E12.5. TH is used to locate sympathetic ganglia. Asterisks in left panels indicate magnified area in middle panels and dashed square indicates magnification area in right panels. E, Magnification of an embryo immunostained for HIF‐2α in a section from trunk axial level of a human embryo at embryonic week 6. Closed arrowheads denote nuclear HIF‐2α staining; open arrowheads denote cytoplasmic HIF‐2α staining

We have previously shown that HIF‐2α is expressed in sympathetic ganglia of human embryos at embryonic week 6.5 (~E12.5 in mice) but that expression is lost in these cells at later stages (fetal week 8).^19^ Here, we detected expression of HIF‐2α positive cells in the dorsal neural tube, as well as in migrating cells in sections through the trunk region of a human embryo of embryonic week ew5 (Carnegie stage 13; Figure 2A). In contrast, there were virtually no HIF‐2α positive cells left within the neural tube at embryonic week ew6 (Carnegie stage 16; Figure 2B). Rather, positive cells could be detected migrating along the ventral pathway followed by sympathoadrenal precursors (Figure 2B). To confirm that that these HIF‐2α positive cells were trunk neural crest cells in human embryos, we co‐stained with HNK‐1 antibody, which is expressed on migrating neural crest cells of human embryos similar to expression in the chick (Figure 2C, cf Figure 1D). This resembled the staining pattern found in chick embryos, but also highlights some differences in the number of positive cells as well as tissues positive for HIF‐2α (compare Figures 1 and 2). These differences likely reflect variation between species as well as the fact that it is difficult to assess exact corresponding developmental stages between them. We further detected HIF‐2α in sympathetic ganglia in mouse embryos at E12.5 by staining adjacent sections for HIF‐2α and TH antibodies, with the latter indicating the location of sympathetic ganglia (Figure 2D). HIF‐2α is a transcription factor that localizes to the nucleus but it has lately also been shown to be expressed in the cytoplasm,^6^, ^9^, ^19^ though its role in the cytoplasm remains unknown. Consistent with this dual localization, we noted HIF‐2α expression in both the nucleus and cytoplasm (Figure 2E), similar to what has been observed in perivascular oxygenated neuroblastoma and glioblastoma cells.^6^, ^20^

### 2.4 Knockdown of HIF‐2α delays embryogenesis and alters gene expression

**FIGURE 3:**
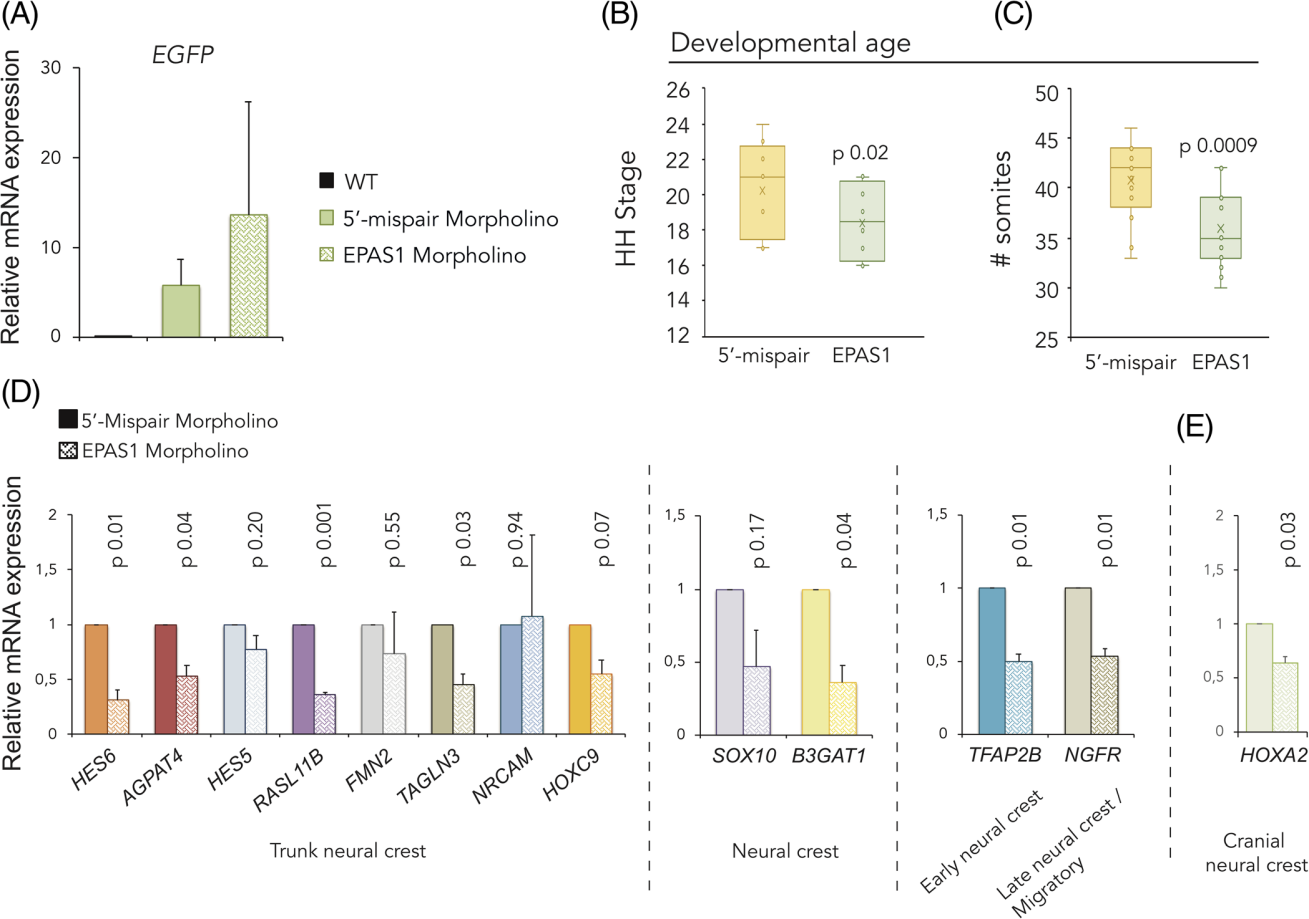
Morpholino mediated knockdown of hypoxia inducible factor (HIF)‐2α delays embryogenesis. A, Relative mRNA expression as measured by qRT‐PCR. WT, wild‐type HH18 embryos. Error bars represent SEM, n = 2 biologically independent replicates. B,C, Determination of developmental age 44 hours postelectroporation with 5′‐mispair or *EPAS1* targeting morpholinos as assessed by head‐ and tail morphology, B, (converted to Hamburger Hamilton (HH) stages. Number of embryos analyzed were n = 20 [5′‐mispair], n = 16 [EPAS1]) or counting somites ex ovo, C, (number of embryos analyzed were n = 17 (5′‐mispair), n = 15 [EPAS1]). Statistical significance was determined by one‐way analysis of variance (ANOVA). D,E, Relative mRNA expression of trunk, D, and cranial, E, neural crest associated genes in dissected neural tube tissue derived from the trunk axial level of embryos electroporated with 5′‐mispair or *EPAS1* morpholinos, measured by qRT‐PCR 24 hours postelectroporation. Data presented as mean of n = 2 biologically independent repeats, error bars denote SEM. Statistical significance was determined by two‐sided student's *t* test

To examine the role of HIF‐2α in vivo, we performed loss‐of‐function experiments in chick embryos using a morpholino‐mediated knockdown approach. Functioning as a surrogate marker, successful electroporation was confirmed by *EGFP* expression (Figure 3A). Experimentally, to ensure that we specifically affected the neural crest and not surrounding tissue such as mesoderm, we injected from the posterior end of the embryo and electroporated the constructs into the lumen of the neural tube. We then let the embryos develop for an additional 24 or 44 to 48 hours (for gene expression and staging/migration assessment, respectively) and analyzed several potentially affected biological processes. Surprisingly, we noticed that HIF‐2α knockdown embryos were developmentally delayed compared with their control counterparts (Figure 3B,C). The stages of embryos following loss of HIF‐2α were determined by their Hamburger and Hamilton developmental stage in ovo (Figure 3B) and by counting somites ex ovo (Figure 3C) 44 hours postinjection. The number of somites was equal on both sides and effects observed were embryo wide.

Knockdown of HIF‐2α further led to decreased expression levels of genes representative of early and migrating neural crest as well as trunk neural crest cells in particular^21^, ^22^ (Figure 3D). The cranial neural crest associated gene *HOXA2* was also slightly downregulated (Figure 3E), though not consistently.

### 2.5 CRISPR/Cas9 mediated knockout of HIF‐2α recapitulates the morpholino phenotype

**FIGURE 4:**
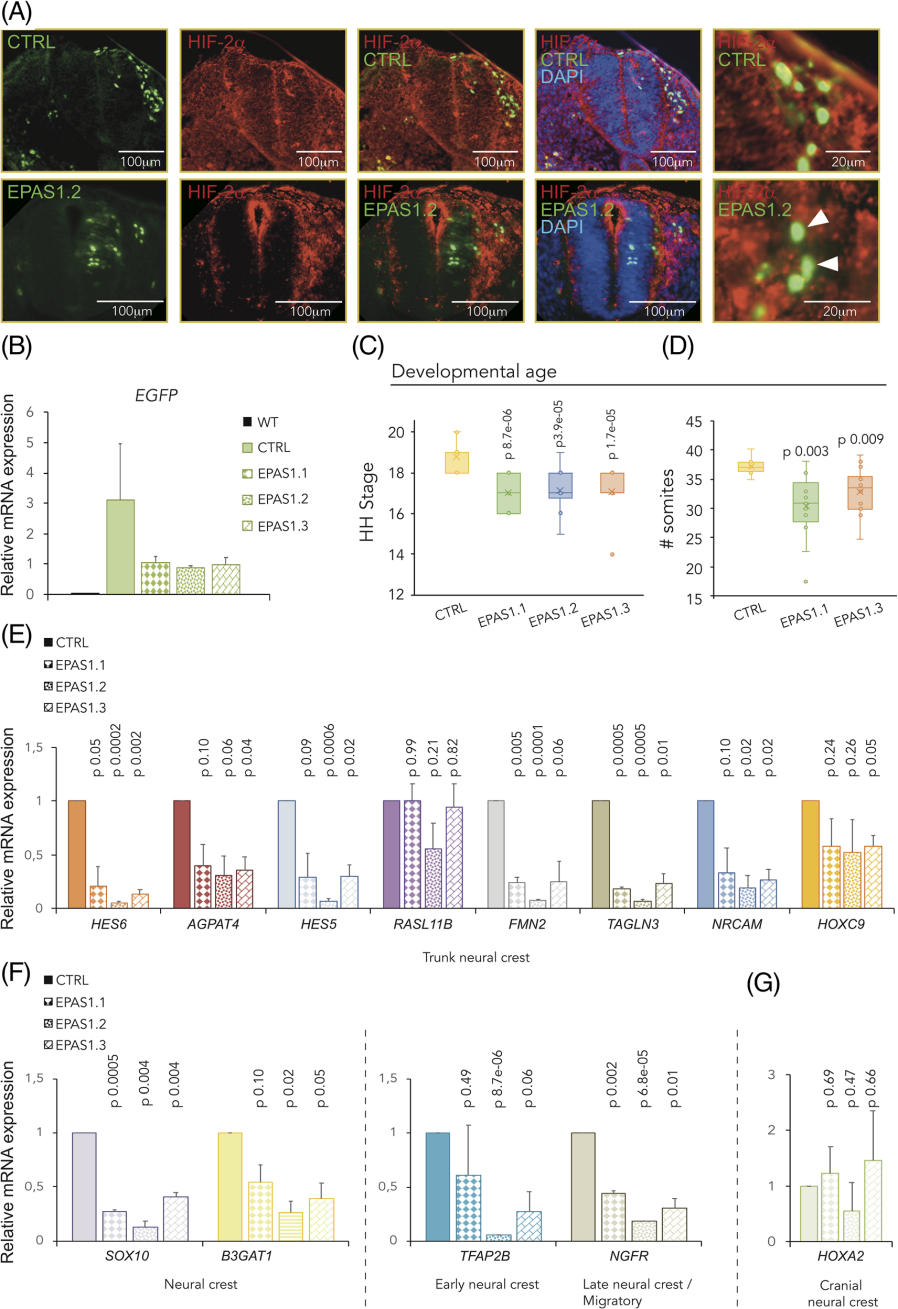
CRISPR/Cas9 mediated knockout of hypoxia inducible factor (HIF)‐2α delays embryogenesis. A, Immunofluorescent staining for HIF‐2α in embryos electroporated with control (CTRL) or HIF‐2α (EPAS1.2) targeting gRNAs. Arrowheads denote GFP+ cells lacking HIF‐2α in knockout embryos. Sections from trunk. B, Relative mRNA expression measured by qRT‐PCR. WT, wild‐type HH18 embryos. C,D, Determination of developmental age 36 hours postelectroporation with a nontargeting (CTRL) gRNA compared to three different gRNAs targeting *EPAS1* (EPAS1.1, EPAS1.2, EPAS1.3) as assessed by head‐ and tail morphology (converted to Hamburger Hamilton [HH] stages, C. Number of embryos analyzed were n = 14 [CTRL], n = 10 [EPAS1.1], n = 14 [EPAS1.2], and n = 14 [EPAS1.3]) or by counting somites ex ovo. (D, Number of embryos analyzed were n = 8 [CTRL], n = 13 [EPAS1.1], and n = 14 [EPAS1.3].) Statistical significance was determined by one‐way analysis of variance (ANOVA), comparing nontargeting CTRL to each individual *EPAS1* gRNA. E‐G, Relative mRNA expression of trunk neural crest, E, neural crest, F, and cranial neural crest, G, associated genes in dissected trunk axial level derived neural tube tissue, measured by qRT‐PCR 36 hours postelectroporation. Data presented as mean of n = 2 biologically independent repeats, error bars denote SEM, B,E‐G. Statistical significance was determined by two‐sided student's *t* test, comparing nontargeting CTRL with each individual *EPAS1* gRNA

Our *EPAS1* morpholino is a splice targeting morpholino, predicted to confer either nonsense‐mediated decay of mRNA or a mutant dysfunctional protein. We could not convincingly detect any changes in HIF‐2α protein expression following morpholino treatment, nor a shift in protein size. This could be explained by other mechanisms‐of‐action for decrease in protein activity or the mosaicism that arises with morpholino treatments in chick embryos. To ensure that the observed biological phenotypes were not due to off‐target effects of our morpholino, we used CRISPR/Cas9 as a second approach to knock out HIF‐2α by designing three different gRNAs targeting *EPAS1* at three different sites. Functional CRISPR mediated knockout of the HIF‐2α protein was demonstrated by immunofluorescence (Figure 4A). The fact that both morpholino and several CRISPR/Cas9 constructs with in total four different target sites within the gene produced the same biological phenotype nicely validates our results and serves as important controls.

After ensuring electroporation efficiency by *EGFP* expression (Figure 4B), we determined the age of the embryos following CRISPR/Cas9 mediated knockout of the protein using head‐ and tail morphology (converted into HH stage; Figure 4C) or by counting somites (Figure 4D) 36 hours postinjection.

### 2.6 Knockdown of HIF‐2α affects cell numbers along the ventral neural crest migratory pathway

**FIGURE 5:**
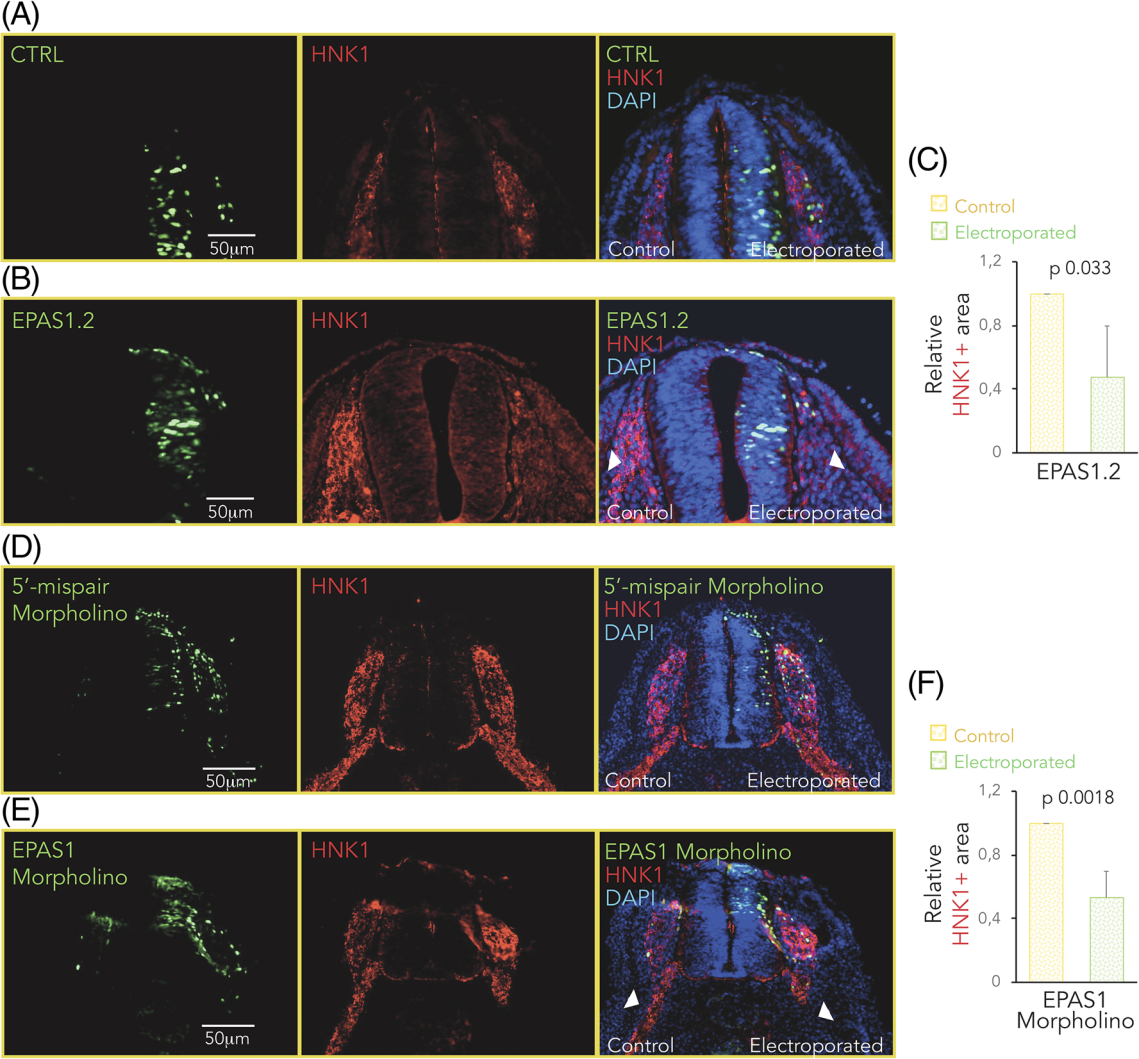
Dysregulation of hypoxia inducible factor (HIF)‐2α expression affects migration of trunk neural crest cells. A‐E, Immunostaining of HNK1 (red) marking migrating crest cells in one‐sided electroporated embryos (right side). Electroporated cells (nontargeting CTRL gRNA, A, gRNA #2 targeting *EPAS1* (EPAS1.2; B), 5′‐mispair morpholino, D, or *EPAS1* morpholino, E) are seen in green. DAPI was used to counterstain nuclei. Embryo sections from trunk axial level are from 36 hours, A,B, or 44 hours, D,E, postelectroporation. Arrowheads highlight the difference in HNK1+ area in control vs electroporated side. C,F, Quantification of area positive for HNK1. Area on electroporated side in EPAS1.2, B, or EPAS1 morpholino, E, embryos was normalized to that of respective control side. Data are presented as mean ± SEM. Statistical significance was calculated using one‐way analysis of variance (ANOVA)

One of the most important features of neural crest cells is their migratory ability. Trunk neural crest cells destined to form the sympathetic chain ganglia migrate ventrally. Following HIF‐2α loss of function using either morpholino or CRISPR/Cas9, HNK1 positive migratory trunk neural crest cells were detected on the control side in all embryos (right panel, left side; Figure 5A‐E) as well as on the side electroporated with nontargeting gRNA CTRL and control 5′‐mismatch morpholino (right panel, left side; Figure 5A,D, respectively). In contrast, loss of HIF‐2α profoundly reduced the number of HNK1 positive cells migrating to ventral regions of the embryo (CRISPR/Cas9, Figure 5B,C; morpholino, Figure 5E,F).

### 2.7 Overexpression of HIF‐2α presents similar effects as loss‐of‐function

**FIGURE 6:**
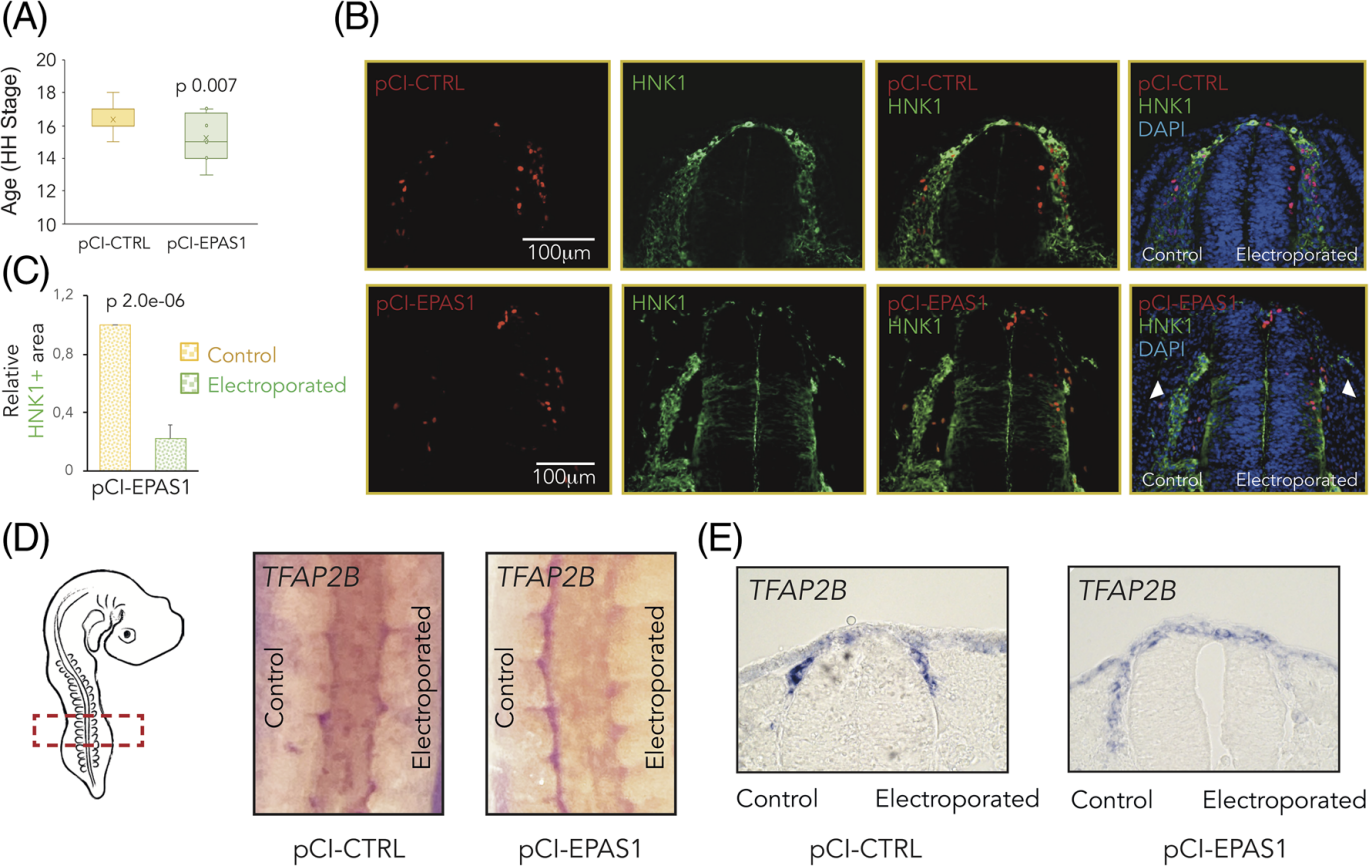
Overexpression of hypoxia inducible factor (HIF)‐2α reflects the knockdown phenotype. A, Hamburger Hamilton (HH) staging of embryos 24 hours postelectroporation with a control (pCI‐CTRL) or *EPAS1* overexpression construct (pCI‐EPAS1), determined by head‐ and tail morphology. Number of embryos analyzed were n = 16 (CTRL), n = 20 (EPAS1). Statistical significance was determined by one‐way analysis of variance (ANOVA). B, Immunostaining of HNK1 (green) marking migrating crest cells in one‐sided electroporated embryos (right side). Electroporated cells (CTRL or EPAS1) are seen in red. DAPI was used to counterstain nuclei. Embryo sections from trunk axial level are taken 48 hours postelectroporation. C, Quantification of area positive for HNK1. Area on electroporated side in pCI‐EPAS1 embryos was normalized to that of respective control side. Data are presented as mean ± SEM. Statistical significance was calculated using one‐way ANOVA. D, In situ hybridization for *TFAP2B* in whole embryos postelectroporation with pCI‐CTRL vs pCI‐EPAS1 constructs. E, Sections at trunk axial level of embryos in, D

Similar to the loss‐of‐function experiments, overexpression of HIF‐2α led to delayed embryonic development (Figure 6A) and perturbed migration as visualized by HNK1 staining (Figure 6B,C). To investigate spatially whether affected genes (Figures 3D and 4E,F) were indeed downregulated in neural crest cells (as indicated by qPCR analyses of gene expression in dissected neural tubes of electroporated embryos), we performed *in situ* hybridization for *TFAP2B* on whole HIF‐2α wild‐type and overexpression embryos. We could detect downregulated levels of *TFAP2B* in delaminated cells on the electroporated side of embryos after overexpression of HIF‐2α, visualized by whole embryo imaging (Figure 6D) and transverse sections (Figure 6E) at trunk axial level.

**FIGURE 7:**
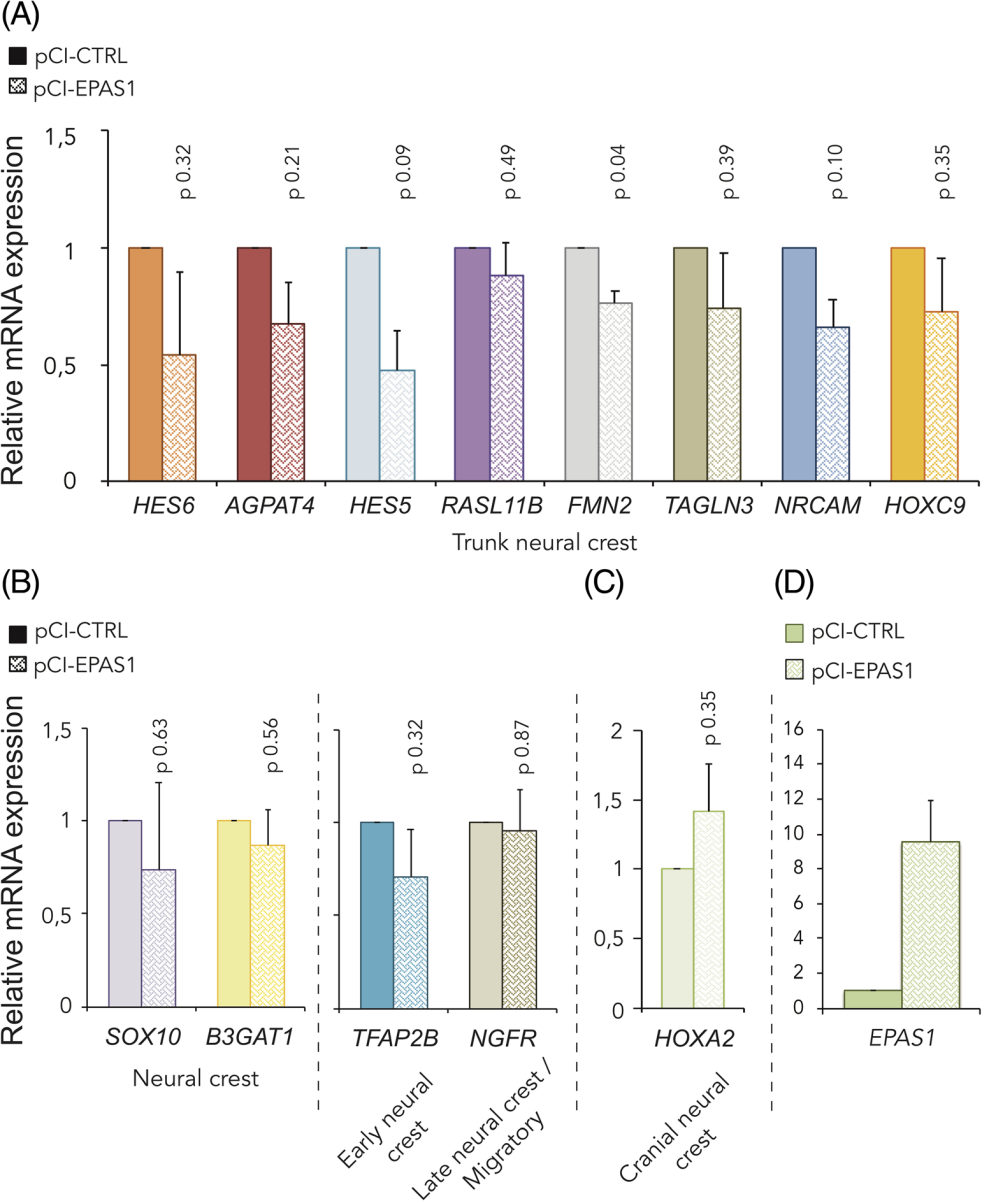
Overexpression of hypoxia inducible factor (HIF)‐2α alters neural crest gene expression. A‐D, Relative mRNA expression of trunk neural crest specific, A, neural crest, B, cranial neural crest, C, and EPAS1, D, genes in dissected neural tube tissue derived from the trunk axial level of embryos electroporated with pCI‐CTRL or pCI‐EPAS1 for overexpression of HIF‐2α, measured by qRT‐PCR 24 hours postelectroporation. Data presented as mean of n = 2 biologically independent repeats, error bars denote SEM. Statistical significance was determined by two‐sided student's *t* test

We also performed qPCR to extend our panel of investigated genes and observed slightly suppressed expression of neural crest‐ and trunk specific genes (Figure 7A,B) whereas expression of cranial neural crest gene *HOXA2* was instead slightly induced (Figure 7C). The less profound effects on neural crest genes from overexpression as compared to knockdown may be attributed HIF‐2α expression level dependent efficiency of the constructs. Overexpression of *EPAS1* was confirmed by qRT‐PCR (Figure 7D).

### 2.8 HIF‐2α knockout does not affect SOX9 distribution

**FIGURE 8:**
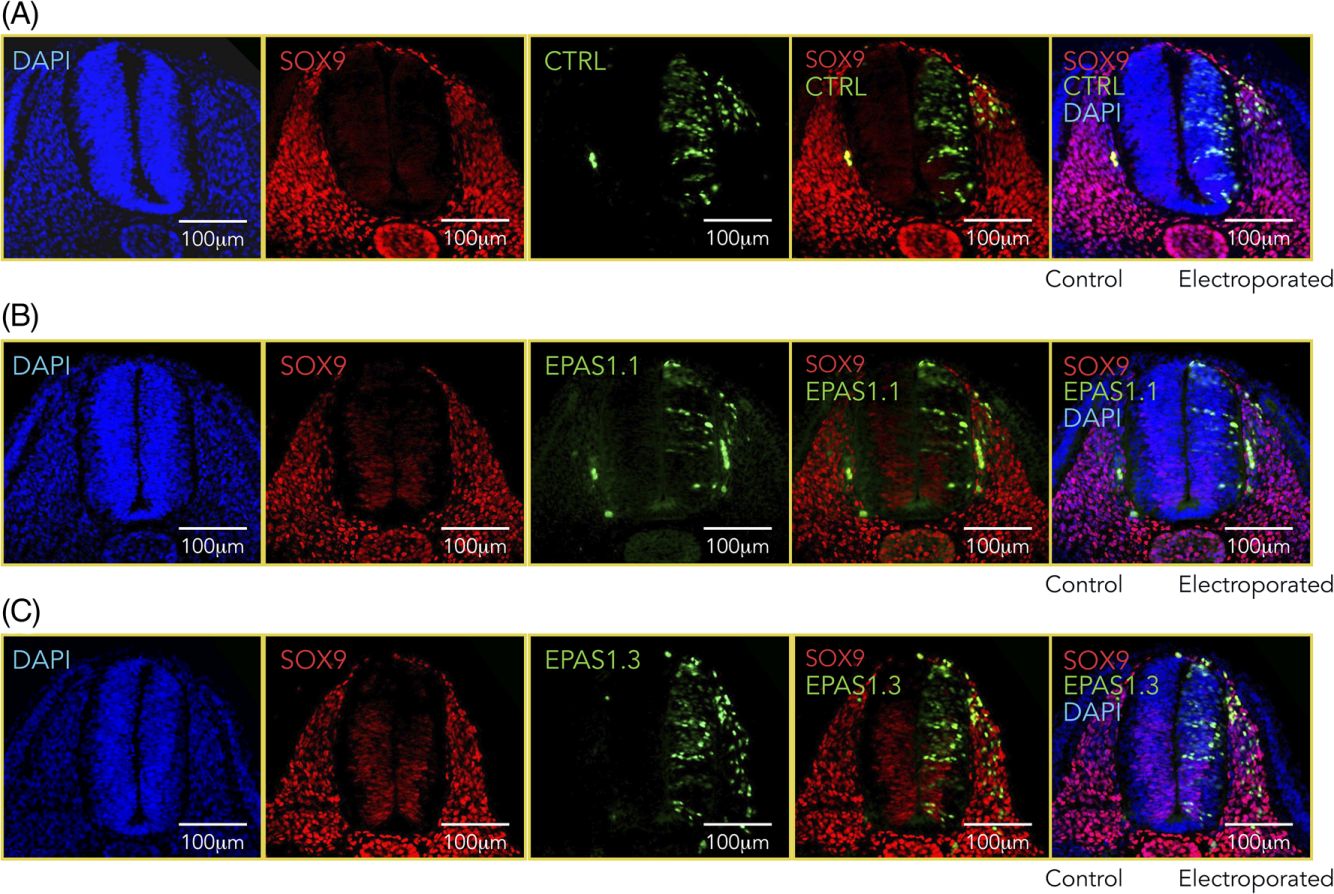
Sox9 expression is not affected by dysregulated levels of Hypoxia inducible factor (HIF)‐2a. A‐C, Immunostaining of Sox9 (red) in one‐sided electroporated embryos (right side). Electroporated cells (nontargeting gRNA [CTRL, A] or gRNA #1 [EPAS1.1, B] and #3 [EPAS1.3, C] targeting EPAS1) are seen in green. DAPI was used to counterstain nuclei. Embryo sections from trunk axial level are from 36 hours postelectroporation

SOX9, a member of the SoxE family of transcription factors, is important for neural crest fate. It is expressed in premigratory neural crest cells at all axial levels and promotes their lineage progression. Importantly, transverse sections through the trunk of embryos electroporated with control (Figure 8A) or two different *EPAS1* targeting gRNA constructs (EPAS1.1 and EPAS1.3, Figure 8B,C, respectively) showed no differences in SOX9 expression. These results suggest that neural crest lineage specification, at least as assessed by SOX9, was unaffected by loss of HIF‐2α.

### 2.9 Trunk neural crest cells proliferate extensively in response to dysregulated HIF‐2α

**FIGURE 9:**
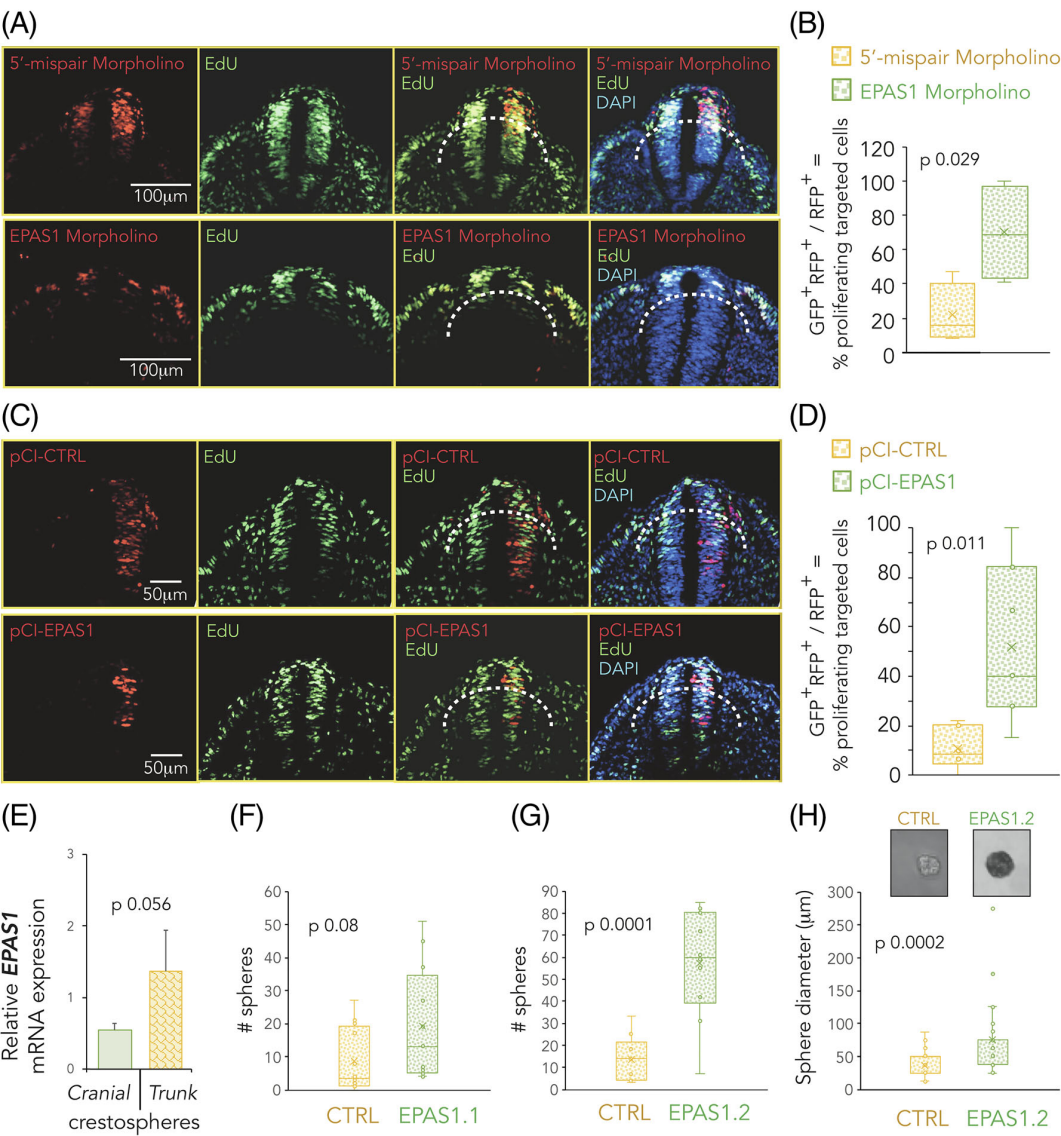
Hypoxia inducible factor (HIF)‐2α affects proliferation and self‐renewal capacity. A‐D, Embryo sections from trunk axial level. Proliferating EdU^+^ cells are green and electroporated cells (morpholinos, A; pCI‐CTRL and pCI‐EPAS1, C) are red. DAPI counterstains nuclei. Only construct targeted neural crest cells (above and outside of dotted line) were quantified (n = 82 [5′‐mispair morpholino] and n = 303 [EPAS1 morpholino], B); n = 211 (pCI‐CTRL) and n = 139 (pCI‐EPAS1), D. Statistical significance calculated using one‐way analysis of variance (ANOVA). E, Relative mRNA expression of *EPAS1* in crestosphere cells established from cranial or trunk axial level measured by qRT‐PCR. Expression is presented as mean of n = 4 (cranial) or n = 3 (trunk) biological replicates and error bars represent SEM. Statistical significance calculated using two‐sided student's *t* test. F,G, Primary sphere assay of crestospheres established from embryos previously electroporated in ovo with non‐ (CTRL) or *EPAS1* (EPAS1.1, F, EPAS1.2, G, targeting gRNAs. One cell/well, n = 10 wells/group). Number of spheres were manually counted after 1 week. Statistical significance was calculated by one‐way ANOVA. H, Size of spheres formed in, G. Manual measurements using the ImageJ software to convert to factual unit (μm). Statistical significance was determined by one‐way ANOVA. Inserted photographs of representative spheres from each group

We next examined cell proliferation in premigratory and early migrating trunk neural crest cells after loss of HIF‐2α using real‐time EdU pulse chase labeling optimized for avian embryos.^23^ Quantifying the proportion of electroporated premigratory and early migrating trunk neural crest cells that had incorporated EdU (by counting RFP^+^ only and RFP^+^/GFP^+^ cells above and outside of the dotted line; Figure 9A) demonstrated a significant increase in proliferating cells with an average proportion of double positive cells of 22% and 70% in the 5′‐mismatch vs EPAS1 morpholino targeted embryos, respectively (*P* .029; Figure 9A,B).

After overexpression of HIF‐2α, real‐time EdU incorporation demonstrated that cells with increased expression of HIF‐2α, similar to HIF‐2α knockdown cells, became highly proliferative with an average proportion of double positive cells of 11% and 52% in the control and HIF‐2α overexpressing embryos, respectively (*P* .011; Figure 9C,D). We conclude that neural crest proliferation, embryonic development and migration is highly sensitive to dysregulated expression of HIF‐2α suggesting that levels must be strictly controlled for proper development (Figures 3B‐E, 4C‐G, 5A‐F, 6A‐E, and 7A‐C).

### 2.10 HIF‐2α downregulation enhances self‐renewal capacity of trunk NC cells

Neural crest‐derived crestosphere cultures^24^, ^25^ enable studies on stemness properties of neural crest cells in vitro. Therefore, we examined *EPAS1* expression in crestosphere cultures, in which multipotent neural crest cells can be maintained in a stem cell‐like state in vitro.^25^, ^26^ Comparing crestosphere cultures derived from trunk vs cranial axial levels (respective axial identities have been extensively characterized in References 25 and 26), showed that *EPAS1* was enriched in trunk crestospheres (Figure 9E).

We further established trunk crestospheres from embryos previously electroporated with a control gRNA construct or two different gRNAs targeting *EPAS1* (EPAS1.1 and EPAS1.2). Primary sphere assays demonstrated that cells with dysregulated HIF‐2α levels had an increased ability to form new spheres when seeded as single cells (1 cell/well; Figure 9F‐G). In addition, crestosphere cultures derived from embryos electroporated with the EPAS1.2 construct formed larger spheres compared to their control counterparts (Figure 9H).

### 2.11 RNA sequencing after loss of HIF‐2α identifies downstream genes associated with invasion, growth arrest, and developmental regulation

**FIGURE 10:**
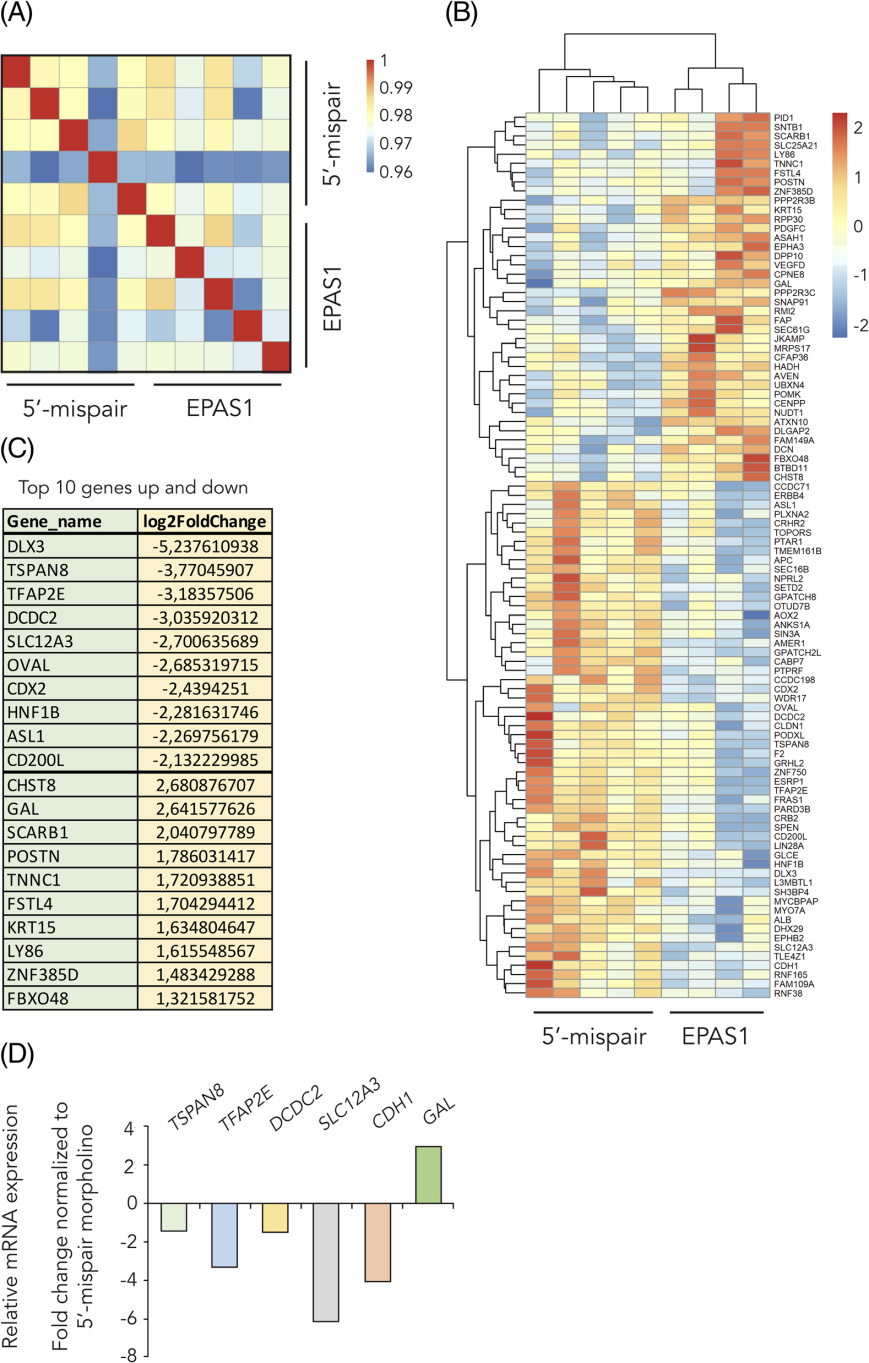
RNA sequencing identifies hypoxia inducible factor (HIF)‐2α downstream genes. A,B, Hierarchical clustering of significantly differentially expressed genes (DEGs; cut‐off *P* < .005) identified from RNA sequencing comparing 5′‐mispair and *EPAS1* morpholino samples. C, List of the top 10 upregulated and top 10 downregulated genes from the RNA sequencing data. D, Relative mRNA expression as measured by qPCR. Samples from Figure 3D,E. EPAS1 morpholino sample was normalized to that of 5′‐mispair control to obtain fold change in expression

To investigate global gene expression changes in cells with dysregulated levels of HIF‐2α, we performed loss of function experiments at premigratory stages of trunk neural crest development (HH10^+^/HH11 in avian embryos) using the splice targeting morpholino as above. Neural tubes from trunk region were dissected 24 hours postelectroporation (at stage ~HH16, when trunk neural crest cells are in the premigratory to early delaminating phase) and subsequently analyzed these by RNA sequencing. Correlation plot of all genes from the dataset demonstrated that HIF‐2α knockdown cells indeed differ from those injected with control scrambled morpholino (spearman *P* > .96; Figure 10A). Setting a cut‐off at *P* < .005 and removing all hits that were not annotated (NA), identified 97 genes of interest (Figure 10B). The top 10 genes downregulated and upregulated (assessed by log2 fold differences in expression) by knockdown of HIF‐2α are summarized in Figure 10C, while the complete list of these 97 genes can be found in Table 1. RNA sequencing results were validated by analyzing selected genes from the top list by qPCR using the samples assessed for neural crest specific gene expression (Figure 3D,E). Genes analyzed by qPCR followed the RNA sequencing predicted effect from HIF‐2α knockdown (Figure 10D).

**TABLE 1:** Full list of the 97 significantly (*P* < .005) DEGs between 5′‐mispair and EPAS1 morpholino samples identified by RNA sequencing

Gene_stable_ID	Gene_name	log2FoldChange	*P*‐value
ENSGALG00000035219	ALB	−1.117182632	.004326406
ENSGALG00000007599	AMER1	−0.405238741	.000373362
ENSGALG00000002723	ANKS1A	−0.620471912	.002221987
ENSGALG00000020876	AOX2	−1.096918485	.00085007
ENSGALG00000000220	APC	−0.47416616	.000273889
ENSGALG00000026364	ASAH1	0.421872055	.002627088
ENSGALG00000002558	ASL1	−2.269756179	.000254935
ENSGALG00000014234	ATXN10	0.477982264	.001858816
ENSGALG00000009642	AVEN	0.319184758	.002524998
ENSGALG00000039595	BTBD11	1.074785502	.000312368
ENSGALG00000040463	CABP7	−1.850580177	.003025143
ENSGALG00000012095	CCDC198	−1.657954928	.00269933
ENSGALG00000006787	CCDC71	−0.470629203	.002673943
ENSGALG00000015395	CD200L	−2.132229985	.000154209
ENSGALG00000000608	CDH1	−1.307331812	.000773978
ENSGALG00000034983	CDX2	−2.4394251	8.25E‐05
ENSGALG00000004687	CENPP	0.424109009	.000169658
ENSGALG00000037504	CFAP36	0.393170817	.00331114
ENSGALG00000004903	CHST8	2.680876707	.004291268
ENSGALG00000026862	CLDN1	−1.995178284	.00010847
ENSGALG00000007025	CPNE8	1.148105385	.004533116
ENSGALG00000001169	CRB2	−0.810451518	.001196479
ENSGALG00000005657	CRHR2	−0.937081004	.004656735
ENSGALG00000042454	DCDC2	−3.035920312	.003127781
ENSGALG00000011274	DCN	0.981364002	.002209936
ENSGALG00000014700	DHX29	−0.444692933	.003215344
ENSGALG00000032937	DLGAP2	1.296643213	.003793901
ENSGALG00000040529	DLX3	−5.237610938	.001107996
ENSGALG00000012156	DPP10	1.123811132	.001713737
ENSGALG00000015403	EPHA3	0.860641014	.001951451
ENSGALG00000004741	EPHB2	−0.418815561	.001893072
ENSGALG00000003126	ERBB4	−1.15424847	.001506512
ENSGALG00000031076	ESRP1	−1.882592181	.001558762
ENSGALG00000008332	F2	−1.908762077	.001551013
ENSGALG00000041153	FAM109A	−1.215286589	.004594595
ENSGALG00000013503	FAM149A	0.797519339	.004470883
ENSGALG00000011099	FAP	1.014306518	.00201386
ENSGALG00000008753	FBXO48	1.321581752	.002069094
ENSGALG00000010316	FRAS1	−0.558639554	.001060929
ENSGALG00000031487	FSTL4	1.704294412	.004365436
ENSGALG00000007047	GAL	2.641577626	.001941927
ENSGALG00000028191	GLCE	−0.598344895	.000897534
ENSGALG00000010350	GPATCH2L	−0.487810446	7.98E‐06
ENSGALG00000041556	GPATCH8	−0.317242944	.003590909
ENSGALG00000037687	GRHL2	−1.807620277	.003321608
ENSGALG00000016124	HADH	0.25603248	.003556043
ENSGALG00000005504	HNF1B	−2.281631746	.00080264
ENSGALG00000012009	JKAMP	0.382527526	.003435274
ENSGALG00000019718	KRT15	1.634804647	.000760169
ENSGALG00000030710	L3MBTL1	−0.530953634	.001495242
ENSGALG00000036022	LIN28A	−1.38546498	.000196313
ENSGALG00000012801	LY86	1.615548567	.003593197
ENSGALG00000002379	MRPS17	0.239466428	.001783389
ENSGALG00000007661	MYCBPAP	−0.506429259	.004333666
ENSGALG00000031450	MYO7A	−0.679727815	.003592535
ENSGALG00000002131	NPRL2	−0.499654709	.003409913
ENSGALG00000004245	NUDT1	0.510897854	.00012546
ENSGALG00000013348	OTUD7B	−0.376188595	.001170227
ENSGALG00000012869	OVAL	−2.685319715	.002991518
ENSGALG00000042645	PARD3B	−0.501189766	1.96E‐06
ENSGALG00000009378	PDGFC	0.796369213	.001886551
ENSGALG00000002963	PID1	1.07251355	.003711941
ENSGALG00000001264	PLXNA2	−0.787868279	.002137423
ENSGALG00000006409	PODXL	−0.886446619	.004450686
ENSGALG00000026210	POMK	0.337221928	.003042971
ENSGALG00000017046	POSTN	1.786031417	.004588475
ENSGALG00000016702	PPP2R3B	0.391820083	.003451236
ENSGALG00000010052	PPP2R3C	0.399540693	.001962875
ENSGALG00000015113	PTAR1	−0.539683505	.001225948
ENSGALG00000010053	PTPRF	−0.480481292	.003964241
ENSGALG00000007155	RMI2	1.140626082	.001382943
ENSGALG00000031018	RNF165	−1.03414841	.001120092
ENSGALG00000015311	RNF38	−0.537721584	.003385081
ENSGALG00000006486	RPP30	0.342750725	.004109447
ENSGALG00000046226	SCARB1	2.040797789	.00121797
ENSGALG00000004424	SEC16B	−1.1085265	.000492445
ENSGALG00000037863	SEC61G	0.557099237	.002778043
ENSGALG00000042051	SETD2	−0.308714867	.000307483
ENSGALG00000004140	SH3BP4	−0.494833722	.001195274
ENSGALG00000001644	SIN3A	−0.319985031	.001157216
ENSGALG00000002957	SLC12A3	−2.700635689	.000223534
ENSGALG00000010117	SLC25A21	0.766964291	.00238367
ENSGALG00000015846	SNAP91	1.1198235	.000304383
ENSGALG00000034528	SNTB1	1.15258033	.003458367
ENSGALG00000036932	SPEN	−0.388461063	.00077178
ENSGALG00000039497	TFAP2E	−3.18357506	.000779405
ENSGALG00000015184	TLE4Z1	−0.444405062	.001437148
ENSGALG00000010896	TMEM161B	−0.546018409	.001173125
ENSGALG00000001459	TNNC1	1.720938851	.003720707
ENSGALG00000020523	TOPORS	−0.473621698	.003381281
ENSGALG00000010152	TSPAN8	−3.77045907	.002947966
ENSGALG00000012259	UBXN4	0.264129274	.004330698
ENSGALG00000043106	WDR17	−1.478943795	.001766045
ENSGALG00000016558	VEGFD	1.266960804	.004105067
ENSGALG00000011283	ZNF385D	1.483429288	.004653997
ENSGALG00000001518	ZNF750	−1.901269846	.002582973

Abbreviation: DEGs, differentially expressed genes.

**FIGURE 11:**
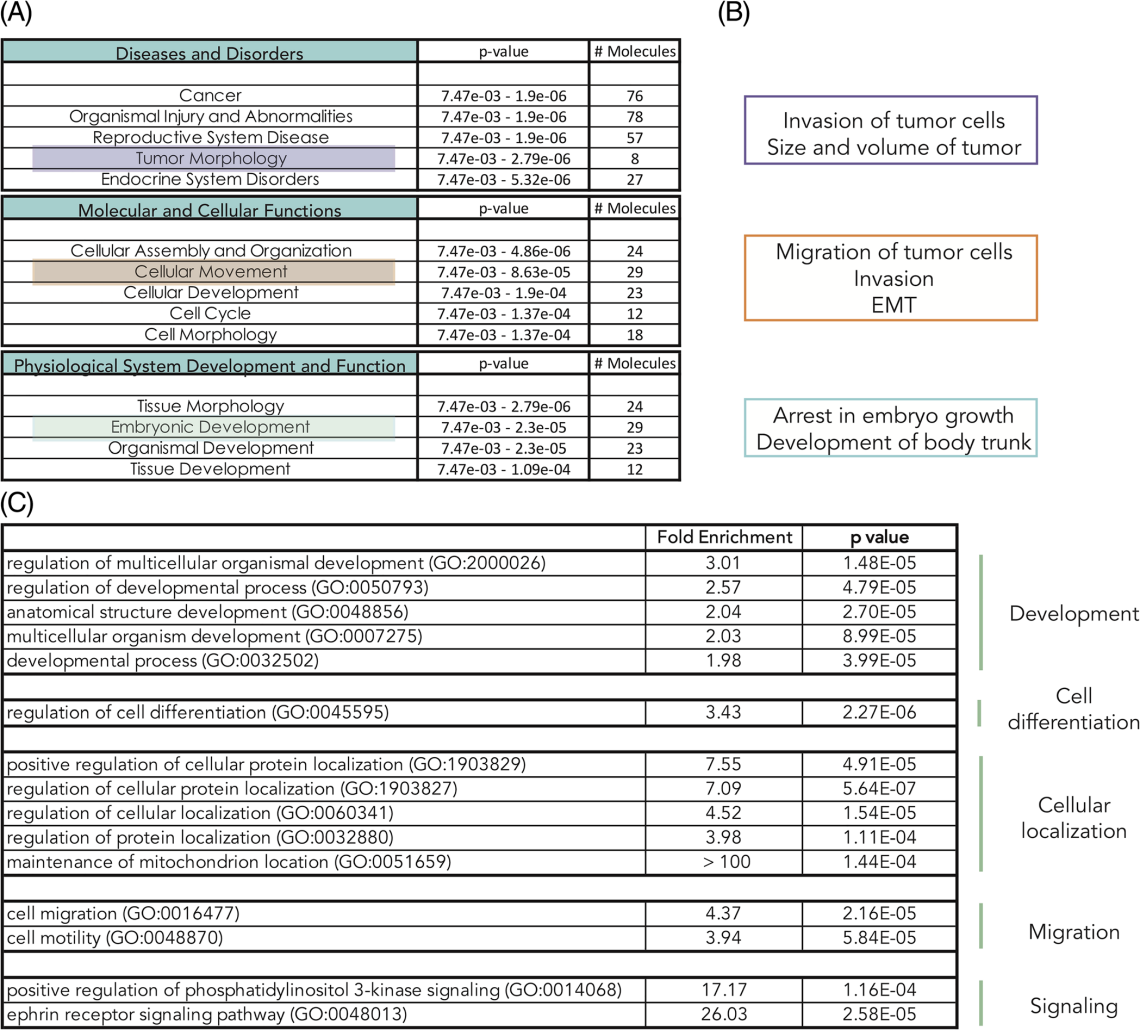
Gene set enrichment analysis identifies hypoxia inducible factor (HIF)‐2α downstream affected processes. A, Top five hits (*P* < .05) in the respective categories “Disease and Disorders,” “Molecular and Cellular Functions,” and “Physiological System Development and Function” identified by hypothesis‐free/exploratory analysis of the 97 differentially expressed genes (DEGs) using IPA (Fishers exact test for the range of *P*‐value calculation). B, Deeper analysis of processes identified in, A. C, Selected list of enriched cellular processes from Panther analyses. Complete list in Table 2

**FIGURE 12:**
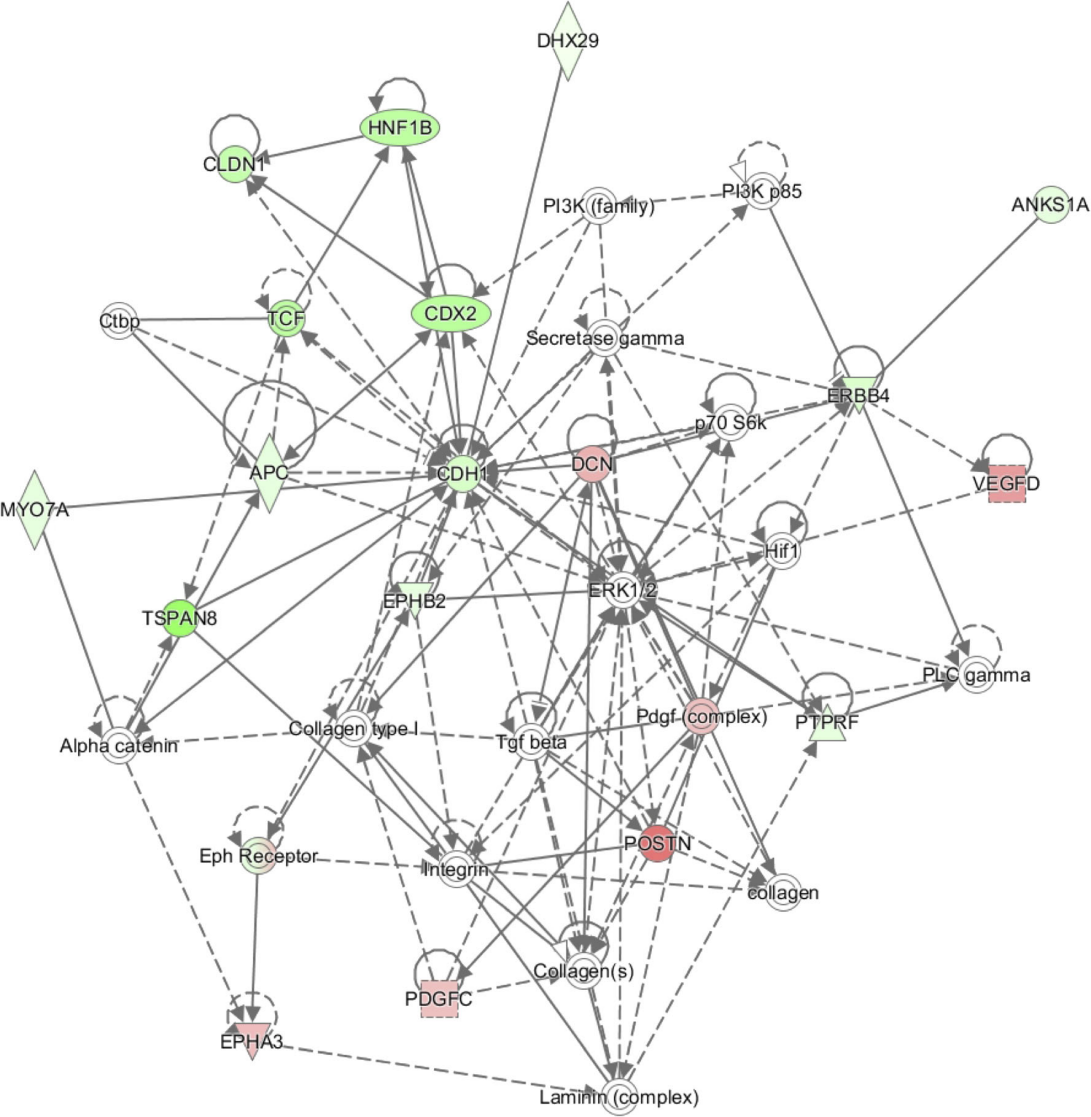
Gene set enrichment analysis identifies key molecules. Top network composed by analyzing significantly differentially expressed genes (DEGs) from RNA sequencing data. The shape of molecules and their meaning, that is, correspondence to protein family, and so forth, is found here: http://qiagen.force.com/KnowledgeBase/KnowledgeIPAPage?id=kA41i000000L5rTCAS. As an example, the diamond‐shaped molecules correspond to enzymes, oval standing shapes should be read as transmembrane receptors, and lying oval shapes are transcription regulators. Green nodes indicate downregulated molecules. The intensity of the color reveals the strength of the expression, that is, the stronger the color the more significant. The dashed lines indicate an indirect interaction between molecules in the network whereas solid lines are direct interactions. The solid arrow explains the direction of the indicated interaction. A line, solid, or dashed, without an arrowhead indicate an RNA‐RNA interaction

Gene set enrichment analysis (GSEA) on the RNA sequencing data demonstrated that two out of the top five processes connected to disease were cancer and tumor morphology (with 29 and 8 out of 97 molecules, respectively; Figure 11A). Deeper analysis of tumor morphology showed that genes associated with invasion of tumor cells and size and volume of tumor were particularly enriched, that is, these associated genes linked to specific disease categories are not due to random chance but are statistically significant (*P* < .05) (Figure 11A,B). Consistent with in vivo data, we identified cellular movement as one of the top molecular and cellular functions affected, with invasion as well as migration of tumor cells and EMT as predicted downstream pathways (Figure 11A,B). GSEA also revealed enrichment of genes associated with embryonic development and in particular arrest in embryo growth (Figure 11A,B). We conclude that the predicted cellular functions derived from our RNA sequencing experiment overlap with in vivo data (cf. Figure 11 with Figures 3, 4, 5, 6, 7, 8, 9). In terms of signaling pathways, top networks from the RNA sequencing data showed enrichment of the ephrin receptor‐ and phosphatidylinositol 3‐kinase (PI3K) signaling pathways (Figures 11C and 12, with full list of gene ontology enriched processes in Table 2). We have previously shown that the PI3K pathway regulates HIF‐2α specifically via mTORC2 and in addition is a promising treatment strategy using a triple PIM/PI3K/mTOR inhibitor in trunk neural crest‐derived tumor form neuroblastoma.^27^, ^28^ Thus, this would be an interesting mechanism to investigate further.

**TABLE 2:** Complete list of processes identified by PANTHER analysis

	Fold enrichment	*P*‐value
Cytolysis by symbiont of host cells (GO:0001897)	> 100	1.44E‐04
Hemolysis in other organism involved in symbiotic interaction (GO:0052331)	> 100	1.44E‐04
Cytolysis in other organism involved in symbiotic interaction (GO:0051801)	>100	2.30E‐06
Maintenance of mitochondrion location (GO:0051659)	>100	1.44E‐04
Trans‐synaptic signaling by trans‐synaptic complex, modulating synaptic transmission (GO:0099557)	>100	1.44E‐04
Hemolysis in other organism (GO:0044179)	>100	1.44E‐04
Hemolysis by symbiont of host erythrocytes (GO:0019836)	>100	1.44E‐04
Killing of cells in other organism involved in symbiotic interaction (GO:0051883)	>100	4.01E‐06
Disruption of cells of other organism involved in symbiotic interaction (GO:0051818)	>100	4.01E‐06
Cytolysis in other organism (GO:0051715)	>100	4.01E‐06
Multiorganism cellular process (GO:0044764)	60.51	3.21E‐05
Cytolysis (GO:0019835)	55.01	4.07E‐05
Disruption of cells of other organism (GO:0044364)	50.43	5.06E‐05
Killing of cells of other organism (GO:0031640)	50.43	5.06E‐05
Axonal fasciculation (GO:0007413)	40.34	8.99E‐05
Neuron projection fasciculation (GO:0106030)	40.34	8.99E‐05
Ephrin receptor signaling pathway (GO:0048013)	26.03	2.58E‐05
Positive regulation of PI3K signaling (GO:0014068)	17.17	1.16E‐04
Positive regulation of cellular protein localization (GO:1903829)	7.55	4.91E‐05
Regulation of cellular protein localization (GO:1903827)	7.09	5.64E‐07
Regulation of cellular localization (GO:0060341)	4.52	1.54E‐05
Cell migration (GO:0016477)	4.37	2.16E‐05
Cell motility (GO:0048870)	3.98	1.11E‐04
Regulation of protein localization (GO:0032880)	3.94	5.84E‐05
Localization of cell (GO:0051674)	3.94	5.84E‐05
Locomotion (GO:0040011)	3.73	2.39E‐05
Regulation of cell differentiation (GO:0045595)	3.43	2.27E‐06
Regulation of response to stimulus (GO:0048583)	3.01	1.48E‐05
Regulation of biological process (GO:0050789)	2.61	3.94E‐05
Regulation of cellular component organization (GO:0051128)	2.57	4.79E‐05
Regulation of multicellular organismal process (GO:0051239)	2.55	1.41E‐05
Positive regulation of cellular process (GO:0048522)	2.33	1.37E‐05
Positive regulation of biological process (GO:0048518)	2.27	1.36E‐05
Negative regulation of cellular process (GO:0048523)	2.25	7.91E‐05
Negative regulation of biological process (GO:0048519)	2.09	5.55E‐06
Cytolysis by symbiont of host cells (GO:0001897)	2.06	2.05E‐06
Regulation of multicellular organismal development (GO:2000026)	2.04	2.70E‐05
Regulation of developmental process (GO:0050793)	2.03	8.99E‐05
Anatomical structure development (GO:0048856)	2.02	6.47E‐05
Multicellular organism development (GO:0007275)	1.98	3.99E‐05
Developmental process (GO:0032502)	1.97	6.32E‐05
Positive regulation of metabolic process (GO:0009893)	1.85	3.35E‐05
Regulation of metabolic process (GO:0019222)	1.47	1.21E‐04
Positive regulation of cellular metabolic process (GO:0031325)	>100	1.44E‐04

Abbreviation: PI3K, phosphatidylinositol 3‐kinase.

### 2.12 HIF‐2α, BMP signaling, and EMT process are predicted upstream regulators of embryo growth

**FIGURE 13:**
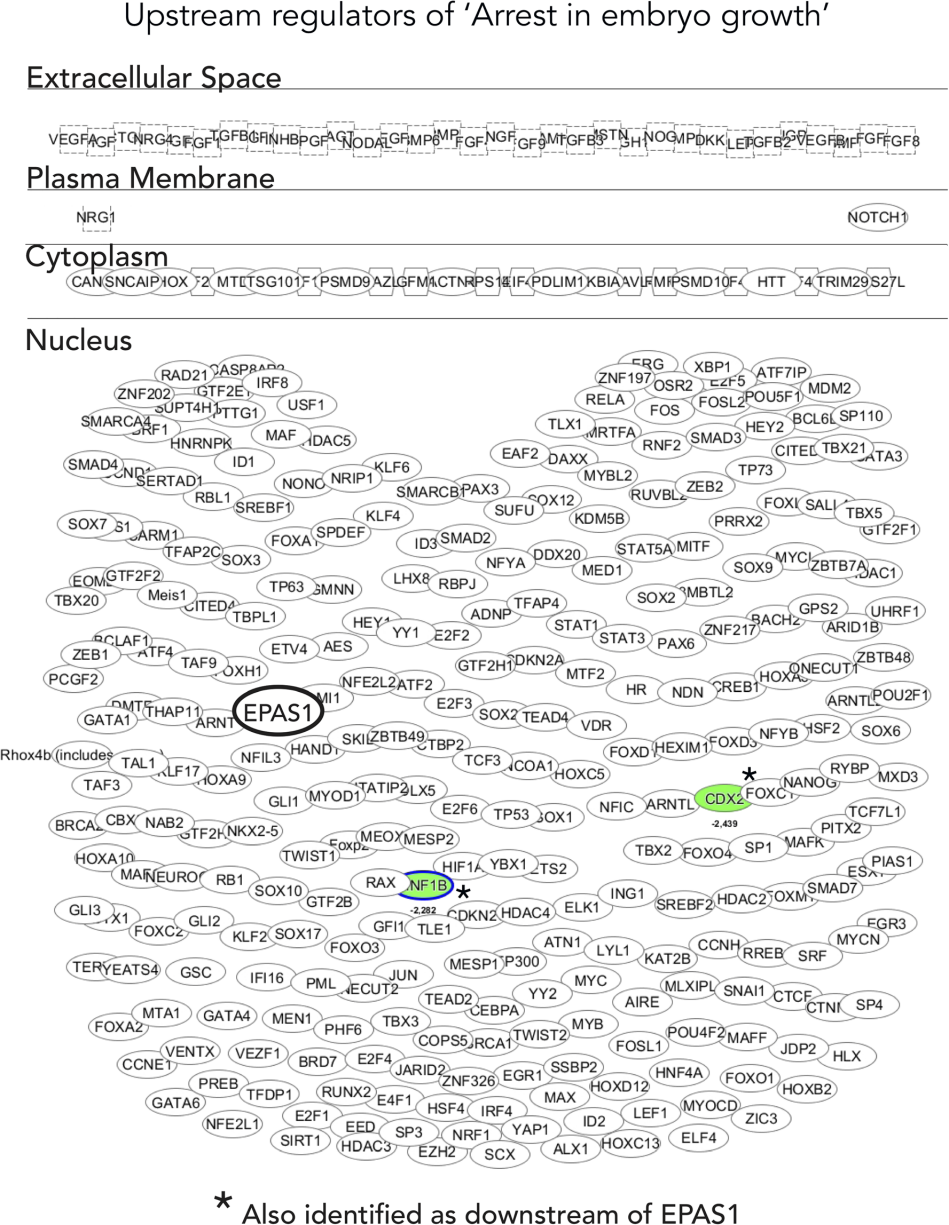
Transcription factor dominance in regulating arrested embryo growth. Deeper analysis of potential upstream regulators of the “arrest in embryo growth” process identified in Figure 11A,B. The shape of molecules and their meaning, that is, correspondence to protein family, and so forth, is found here: http://qiagen.force.com/KnowledgeBase/KnowledgeIPAPage?id=kA41i000000L5rTCAS. As an example, the diamond‐shaped molecules correspond to enzymes, oval standing shapes should be read as transmembrane receptors and lying oval shapes are transcription regulators. Green nodes indicate downregulated molecules. The intensity of the color reveals the strength of the expression, that is, the stronger the color the more significant

Given the effects we observed on embryonic development in vivo, we mapped potential upstream regulators of arrest in embryo growth (one of the identified top processes by RNA sequencing data). As expected, most genes were transcription factors localized in the nucleus (Figure 13). Connecting and validating the in vivo data and RNA sequencing downstream analyses, *EPAS1* itself was identified as one of the upstream genes regulating this process (Figure 13). Further, among the predicted upstream regulators of arrested growth, genes associated with stem cells, BMP signaling, and EMT were highly enriched (Tables 3 and 4).

**TABLE 3:** Selected genes identified as potential upstream regulators of arrested embryo growth. Genes associated with stem cells, BMP signaling, and EMT were particularly enriched

(a) Stem cell associated genes		
Upstream regulator	Molecule type	*P*‐value of overlap
SOX2	Transcription regulator	3,72E‐16
POU5F1/OCT4	Transcription regulator	5,29E‐16
E2F4	Transcription regulator	2,66E‐12
KLF4	Transcription regulator	2,61E‐11
NANOG	Transcription regulator	2,81E‐07
EZH2	Transcription regulator	2,69E‐08
GLI1	Transcription regulator	1,68E‐05
NOTCH1	Transcription regulator	2,31E‐03
KLF2	Transcription regulator	3,00E‐03
SALL4	Transcription regulator	1,67E‐02
HEY1	Transcription regulator	1,97E‐02
KLF6	Transcription regulator	2,66E‐02
HEY2	Transcription regulator	3,57E‐02
BMI1	Transcription regulator	2,84E‐04

(b) BMP signaling associated genes		
Upstream regulator	Molecule type	*P*‐value of overlap
BMP4	Growth factor	5,74E‐11
BMP2	Growth factor	2,69E‐03
BMP10	Growth factor	5,06E‐03
BMP6	Growth factor	1,17E‐02
SMAD2	Transcription regulator	5,66E‐09
SMAD7	Transcription regulator	8,82E‐06
SMAD4	Transcription regulator	5,43E‐05
SMAD3	Transcription regulator	1,38E‐03

(c) EMT associated genes		
Upstream regulator	Molecule type	*P*‐value of overlap
SNAI1	Transcription regulator	8,22E‐04
ZEB2	Transcription regulator	1,44E‐03
TWIST1	Transcription regulator	3,00E‐03
ZEB1	Transcription regulator	1,10E‐02
LEF1	Transcription regulator	2,03E‐02
NODAL	Growth factor	2,13E‐02

Abbreviation: EMT, epithelial‐to‐mesenchymal transition.

**TABLE 4:** Complete list of genes identified as potential upstream regulators of arrested embryo growth

Upstream of arrest in embryo growth			
Upstream regulator	Molecule type	*P*‐value of overlap	Target molecules in dataset
MYC	Transcription regulator	1.40E‐22	ABCA1,ACACA,ACVR1,ACVR2A,AMD1,ATF2,CASP8,CDK2,CDK4,CDK6,CDKN2A,CDX2,COMMD3‐BMI1,CUL1,DDX11,DLX3,DNMT1,EOMES,EZH2,F2,FOXA2,G6PD,GATA4,GCLC,HAND1,HIF1A,KAT2A,KRAS,LIMS1,Macf1,MAX,MCL1,METAP2,MGAT1,MYCN,OTX2,PARP1,PNO1,RAB10,RAD51,RB1,SLC25A19,SMAD4,SUMO2,TDG,TDGF1,TLN1,TP53,TXN,TXNRD1,WLS
SOX2	Transcription regulator	3.72E‐16	ACVR1,ACVR2A,CDKN2A,CDX2,COMMD3‐BMI1,DLX3,DNMT1,EOMES,FGF4,FLT1,FOXA2,GATA4,GSC,HAND1,ISL1,Macf1,MAX,MYCN,NOTCH1,OTX2,SMAD4,SNAI1,TDGF1,TXNRD1,WLS
POU5F1	Transcription regulator	5.29E‐16	ACVR1,ACVR2A,CDX2,DLX3,DNMT1,EOMES,FGF4,FOXA2,GATA4,GSC,HAND1,HIF1A,ISL1,Macf1,MAX,MCL1,MYCN,PARP1,SMAD4,SNAI1,TDGF1,TP53,TXNRD1,WLS,ZEB2
RB1	Transcription regulator	1.67E‐14	ATF2,BECN1,CASP8,CDK2,CDKN2A,CDX2,CHAF1A,DDX11,DNMT1,EOMES,EZH2,FLT1,MCM10,MTOR,MYL1,PARP1,RAD51,RB1,RBL2,SDHD,SMARCA5,TOPBP1,TP53,TUBG1,ZEB2
E2F1	Transcription regulator	5.03E‐13	BECN1,CASP8,CDK2,CDK4,CDKN2A,DDX11,DNMT1,EZH2,FLT1,GINS1,HSP90B1,KRAS,MCL1,MCM10,MYCN,PIK3C3,RAD51,RB1,RBBP8,REV3L,SMARCA5,TOPBP1,TP53,TXNRD1,UHRF1,XRCC1
E2F4	Transcription regulator	2.66E‐12	BECN1,CDK2,DDX11,GINS1,HSP90B1,MCL1,MCM10,MYCN,NASP,RAD51,RB1,RBBP8,RBL2,SHH,SMARCA5,TOPBP1,TP53,UHRF1
TGFB1	Growth factor	4.38E‐12	ABCA1,ACVR1,ACVRL1,AMD1,APLNR,BECN1,CASP8,CDC7,CDH5,CDK2,CDK4,CDKN2A,CTCF,DNMT1,EHMT2,EOMES,F2,F2RL1,FLT1,FOXA2,GCLC,GNA13,HIF1A,KRAS,LDB1,LIMS1,MAPK7,MYCN,NOC3L,NOTCH1,PCGF2,PIK3C3,PNO1,PSMC3,RAC1,RAD51,RASA1,RB1,RBL2,SMAD2,SMAD4,SNAI1,SOX4,THBD,TP53,TXNRD1,VCL,ZEB2
KLF4	Transcription regulator	2.61E‐11	ACVR1,ACVR2A,CDX2,DLX3,EOMES,FLT1,GATA4,HAND1,Macf1,NF1,NOTCH1,OTX2,SMAD4,TDGF1,THBD,TP53,TXNRD1,WLS
BMP4	Growth factor	5.74E‐11	ACVR2A,ACVR2B,CDH5,CDK2,CDKN2A,CDX2,DLX3,MYCN,PTCH1,SHH,SNAI1,SPINT1,TAL1,TBX5,TDGF1,TP53
TP63	Transcription regulator	3.81E‐10	ATF2,BECN1,CDK6,CDKN2A,DICER1,DNMT1,FOXA2,G6PD,HIRA,IHH,MCM10,NOTCH1,RAD51,RAD9A,RBBP8,SMAD2,SMAD4,SNAI1,TP53,ZEB2
EGF	Growth factor	1.75E‐09	ARC,CDK2,CDK4,CDKN2A,CDX2,CTCF,FLT1,FOXA2,HIF1A,HIRA,KRAS,MCL1,MYCN,NOTCH1,Nrg1,RAC1,SNAI1,SOX4,TBP,TP53,TRPM7,VCL
HDAC1	Transcription regulator	2.55E‐09	ARC,CDK2,CDKN2A,COMMD3‐BMI1,EHMT2,FLT1,MCM10,NASP,PCYT1A,SLC8A1,SNAI1,TAL1,TBX5,TP53,UHRF1,USP7
FOXM1	Transcription regulator	3.15E‐09	ATF2,CDK2,CDKN2A,COMMD3‐BMI1,FLT1,MYCN,PLK4,PTCH1,SNAI1,TP53,XRCC1,ZEB2
RNF2	Transcription regulator	3.34E‐09	CDKN2A,CDX2,COMMD3‐BMI1,EOMES,FOXA2,GATA4,HAND1,PCGF2,TP53
GMNN	Transcription regulator	3.98E‐09	ACVR1,ACVR2A,CDX2,EOMES,HAND1,Macf1,SMAD4,TP53,TXNRD1,WLS
TP53	Transcription regulator	5.56E‐09	CASP8,CDC7,CDK2,CDK4,CDKN2A,DICER1,DLD,DNMT1,EZH2,FOXA2,FXN,G6PD,GATA4,GNA13,HIF1A,HTT,LIAS,MCL1,MGAT1,NOTCH1,PARP2,PCGF2,PIK3C3,PNO1,PTCH1,PTPN11,RAD50,RAD51,RAF1,RB1,RBL2,SDHD,SNAI1,TBP,TBX5,TDG,THBD,TLN1,TP53,TSG101,UHRF1,VCL,ZEB2
SMAD2	Transcription regulator	5.66E‐09	BECN1,CDH5,CDKN2A,CDX2,FLT1,GSC,RAC1,SMAD2,SMAD4,SNAI1
SOX1	Transcription regulator	1.98E‐08	ACVR1,ACVR2A,CDX2,EOMES,HAND1,Macf1,SMAD4,TXNRD1,WLS
SP110	Transcription regulator	2.23E‐08	ATF2,DICER1,FLII,GNA13,MCL1,MYCN,PLCG1,RAC1,RARRES2,SMAD2,SOX4
EZH2	Transcription regulator	2.69E‐08	CDK4,CDK6,CDKN2A,CDX2,CUL1,DNMT1,EOMES,EP300,EZH2,FGF4,FLT1,GATA4,KRAS,RNASEH1,SHH,SNAI1,TP53
RBL1	Transcription regulator	2.84E‐08	CDK2,CDKN2A,MCM10,MTOR,MYCN,NOTCH1,RB1,RBL2,TP53
PCGF2	Transcription regulator	2.97E‐08	CDK4,CDK6,CDKN2A,CDX2,GATA4,NOTCH1,TP53
AGT	Growth factor	3.31E‐08	BECN1,CASP8,CDK4,CDKN2A,FLT1,HIF1A,KRAS,MAPK7,PARP1,RAC1,RIPK3,SLC8A1,SOX4,TBX20,TBX5,THBD,TP53,TRPM7,ZEB2
SOX3	Transcription regulator	3.58E‐08	ACVR1,ACVR2A,CDX2,EOMES,HAND1,Macf1,SMAD4,TXNRD1,WLS
E2F3	Transcription regulator	7.82E‐08	BECN1,CDK2,CDKN2A,DAG1,EZH2,KRAS,MCM10,MYCN,RAD51,RB1,THBD,TOPBP1
HGF	Growth factor	1.55E‐07	ATF2,CASP8,CDK2,CDK4,CDKN2A,DDX11,FLT1,FOXA2,GNA13,MCL1,Nrg1,PLK4,RAC1,RAD50,RARRES2,SNAI1,THBD,TOPBP1,TP53
NANOG	Transcription regulator	2.81E‐07	CDK6,EOMES,FOXA2,GATA4,GSC,ISL1,MAX,MYCN,SRRT,TP53
E2F2	Transcription regulator	2.83E‐07	BECN1,CDKN2A,EZH2,MCM10,MYCN,RAD51,TOPBP1,TP53
PTTG1	Transcription regulator	3.89E‐07	HIF1A,PARP2,RAD50,RAD9B,REV3L,SNAI1,TDG,TP53
EIF4E	Translation regulator	5.63E‐07	CDK2,CDK4,CDKN2A,EP300,HIF1A,MCL1,NRAS,RB1,SNAI1,TP53
VEGFA	Growth factor	5.73E‐07	CDH5,CDKN2A,EOMES,FLT1,HIF1A,LDB1,MCL1,MYCN,NOTCH1,PARP1,SDHD,TAL1,THBD,TP53
SRF	Transcription regulator	5.80E‐07	AMD1,ARC,CDK4,FLT1,GATA4,MCL1,MYL1,RAF1,SHH,SLC8A1,SNX2,TAL1,TBP,TLN1,VCL
HNF4A	Transcription regulator	7.49E‐07	ACVR1,APH1A,ATF2,ATF7,CDK2,DAG1,DLX3,FOXA2,G6PD,HIF1A,HNF1B,HSP90B1,KAT7,LDB1,LIMS1,MAPK7,METAP2,NOC3L,NRAS,OTX2,PAGR1,PCGF2,PCYT1A,PELO,PIK3C3,PNO1,PSMC4,PTPN11,RAB10,RAD50,RAD51,RASA1,RBL2,SHH,SLC25A19,SLC33A1,SMAD4,SMARCA5,TLN1,TSG101,TXN,TXNRD1
SP1	Transcription regulator	9.58E‐07	ABCA1,ACVRL1,CDK2,CDK4,CDK6,CDKN2A,DLX3,FLT1,HIF1A,MCL1,MYCN,NF1,PARP1,PCYT1A,RB1,THBD,TP53,TXNRD1,ZEB2
FOXO3	Transcription regulator	1.55E‐06	ACVR1,BECN1,CASP8,CDH5,CDK4,GCLC,HIF1A,MAX,RASA1,RBL2,SMAD4,SNAI1,TAL1,TP53
INHBA	Growth factor	1.63E‐06	ACVR1,ACVR1B,ACVR2A,ACVR2B,CDK4,FOXA2,KRAS,MCL1,SMAD2,SOX4,TAL1
NKX2‐5	Transcription regulator	1.75E‐06	ACACA,BMP10,GATA4,HAND1,MYCN,TBX5
CCND1	Transcription regulator	2.29E‐06	CDK2,CDK4,CDK6,CDKN2A,DNMT1,GATA4,MCM10,NOTCH1,PCYT1A,RAD51,RB1,SOX4,TP53,UHRF1
TBX20	Transcription regulator	2.89E‐06	BMP10,MYCN,TBX5
HNRNPK	Transcription regulator	3.07E‐06	ATF2,FGF4,G6PD,HUS1,RASA1,RB1
CDKN2A	Transcription regulator	3.20E‐06	AMD1,CDK2,CDKN2A,CHAF1A,CUL1,EZH2,GNA13,KRAS,MCL1,MYCN,RB1,TP53,TSG101
PML	Transcription regulator	3.48E‐06	ACACA,CDK2,CDKN2A,SNAI1,SUMO2,TOPBP1,TP53,TXN,TXNRD1
ETS1	Transcription regulator	4.81E‐06	CDH5,CDK11A,CDK2,CDK6,CDKN2A,FLT1,MCL1,PARP1,SNAI1,TP53,ZEB2
CTNNB1	Transcription regulator	6.93E‐06	ACVR2A,CDKN2A,COMMD3‐BMI1,EOMES,FOXA2,GNA12,IHH,ISL1,MCL1,MYCN,NOTCH1,PTCH1,SHH,SNAI1,SOX11,SOX4,TBX20,TBX5,TDGF1,TP53,ZEB2
NCOA1	Transcription regulator	7.48E‐06	ABCA1,CASP8,EOMES,HIF1A,HIRA,LIAS,PDCD2,POU2F1,RB1
CITED2	Transcription regulator	8.06E‐06	CDKN2A,CDX2,COMMD3‐BMI1,HIF1A,PCGF2
SMAD7	Transcription regulator	8.82E‐06	ACVR1,ACVR1B,ACVR2A,ACVR2B,BMPR2,FOXA2,GATA4,RAC1,TXN
MED1	Transcription regulator	1.41E‐05	ACACA,ARID1A,CDK2,CDK4,CHAF1A,MCL1,MYCN,NF1,TP53
TEAD4	Transcription regulator	1.50E‐05	CDX2,EOMES,FLT1,GATAD2A,HIF1A
GLI1	Transcription regulator	1.68E‐05	ARC,CDK2,COMMD3‐BMI1,FOXA2,GATA4,MYCN,PTCH1,SNAI1,TP53,USP7
FGF2	Growth factor	1.99E‐05	ARC,CDX2,FLT1,HIF1A,HIRA,KRAS,MYCN,NF1,NOTCH1,RAF1,RB1,SHH,SNAI1,TP53
STAT3	Transcription regulator	2.24E‐05	BECN1,CDH5,CDKN2A,COPS5,DNMT1,EOMES,FLT1,HIF1A,IHH,MCL1,NOTCH1,POU2F1,SHH,SNAI1,THBD,TP53,USP7
CCNH	Transcription regulator	2.26E‐05	CDX2,COMMD3‐BMI1,GATA4,HAND1
EED	Transcription regulator	2.92E‐05	CDKN2A,CDX2,GATA4,SHH,TBX5
SIRT1	Transcription regulator	3.16E‐05	ABCA1,ATF7,CDKN2A,EP300,GATA4,HIF1A,MGAT1,NF1,PARP1,RAC1,TAL1,THBD,TP53
HDAC2	Transcription regulator	3.22E‐05	CDK2,CDKN2A,COMMD3‐BMI1,MCM10,MYCN,NASP,SLC8A1,TP53
ID1	Transcription regulator	4.10E‐05	CDKN2A,FOXA2,MESP1,NOTCH1,PCGF2,SNAI1
EIF4G1	Translation regulator	4.17E‐05	HIF1A,RAD50,RAD51,TP53
NRG1	Growth factor	4.36E‐05	ABCA1,CDX2,DAG1,GATA4,HIF1A,MCL1,NOTCH1,SNAI1,SOX4,VCL
FOXO1	Transcription regulator	4.46E‐05	ACACA,CASP8,CDH5,CDKN2A,EOMES,FOXA2,HIF1A,HUS1,MYCN,PIK3C3,RBL2,SLC25A19,SMAD4,TP53
YAP1	Transcription regulator	4.50E‐05	CASP8,CDK6,CDX2,DICER1,RAD51,SMAD2,UHRF1
FGF7	Growth factor	4.82E‐05	CDK2,CDK4,CDKN2A,Nrg1,SHH,TP53
SMAD4	Transcription regulator	5.43E‐05	CDH5,CDKN2A,MAPK7,MTOR,MYCN,RAC1,SHH,SMAD2,SMAD4,SNAI1
MDM2	Transcription regulator	5.63E‐05	CDK4,CDKN2A,HIF1A,MYCN,TP53,TSG101
SHOX	Transcription regulator	5.87E‐05	RB1,RBL2,TP53
PSMD10	Transcription regulator	5.98E‐05	CDK2,CDK4,HIF1A,TP53
CEBPA	Transcription regulator	6.27E‐05	ACACA,APLNR,CDK4,COPS5,DLX3,FLT1,FOXA2,HNF1B,KDM1A,MYCN,OTX2,RARRES2,THBD,VCL
SREBF2	Transcription regulator	6.54E‐05	ABCA1,ACACA,G6PD,PCYT1A,PTCH1,RARRES2
GATA6	Transcription regulator	6.82E‐05	BMPR2,CDX2,DLD,DLX3,FOXA2,GATA4,HNF1B,OTX2,SHH
HAND1	Transcription regulator	7.07E‐05	ACACA,FLT1,HAND1,NOTCH1
CCNE1	Transcription regulator	8.29E‐05	CDK2,HIF1A,PCYT1A,TP53
GATA4	Transcription regulator	9.31E‐05	ACACA,BECN1,CDX2,DLX3,GATA4,MYL1,OTX2,SLC8A1,TAL1
IRF4	Transcription regulator	9.38E‐05	CDK2,CDK6,CDKN2A,EOMES,FLT1,RAC1,RAD51,XRCC1
TLX1	Transcription regulator	1.12E‐04	MCL1,RAD51,RAF1,TAL1
TEAD2	Transcription regulator	1.14E‐04	RAD51,TP53,UHRF1
RUNX2	Transcription regulator	1.18E‐04	CSNK2B,HIF1A,IHH,ISL1,SHH,SNAI1,TLN1
ARNT	Transcription regulator	1.24E‐04	CDK4,FLT1,G6PD,HIF1A,Macf1,SHH,TP53
PAX6	Transcription regulator	1.35E‐04	CDK2,CDK6,EOMES,F2,ISL1,OTX2,SMAD2,SMAD4,WLS
PITX2	Transcription regulator	1.37E‐04	ATF2,EOMES,FOXA2,ISL1,SNAI1,TBX5,ZEB2
ELK1	Transcription regulator	1.51E‐04	BMPR2,CDKN2A,GRK2,MCL1,SNAI1
ZIC3	Transcription regulator	1.95E‐04	FOXA2,GATA4,TBX5
BRCA1	Transcription regulator	2.06E‐04	EP300,NOTCH1,POU2F1,RAD51,RB1,RBL2,TBP,TP53
YEATS4	Transcription regulator	2.43E‐04	CDKN2A,TP53
TERF1	Transcription regulator	2.43E‐04	CDKN2A,TP53
ELF4	Transcription regulator	2.43E‐04	CDKN2A,DLX3,PIK3C3,TP53
MYCN	Transcription regulator	2.66E‐04	ABCA1,CDH5,COMMD3‐BMI1,EZH2,FOXA2,GATA4,MYCN,SLC25A19,TP53,ZEB2
TFDP1	Transcription regulator	2.73E‐04	CASP8,CDKN2A,MYCN,TP53
BMI1	Transcription regulator	2.84E‐04	CASP8,CDKN2A,CUL1,DNMT1,TP53
EGR1	Transcription regulator	3.11E‐04	ARC,CASP8,FLT1,HIF1A,RB1,SHH,SNAI1,TP53
GLI2	Transcription regulator	3.50E‐04	DLX3,FOXA2,GATA4,MYCN,PTCH1,SNAI1
MESP1	Transcription regulator	3.74E‐04	GATA4,SNAI1,ZEB2
MAFG	Transcription regulator	3.74E‐04	GCLC,TP53,TXNRD1
GLI3	Transcription regulator	3.77E‐04	COMMD3‐BMI1,FOXA2,MYCN,PTCH1,SHH
TGFB2	Growth factor	4.08E‐04	ABCA1,CDH5,CDKN2A,NOTCH1,OSR1,SMAD2
IGF1	Growth factor	4.31E‐04	ABCA1,ACACA,CDK2,CDK4,CDKN2A,HIF1A,IHH,KAT2A,MCL1,MYCN,PARP1,TP53,UBTF
MESP2	Transcription regulator	4.51E‐04	GATA4,SNAI1,ZEB2
LYL1	Transcription regulator	5.37E‐04	CDH5,RAPGEF2,TAL1
JARID2	Transcription regulator	5.37E‐04	CDX2,HAND1,NOTCH1
RELA	Transcription regulator	5.71E‐04	BECN1,CASP8,CDKN2A,CTCF,EOMES,HIF1A,HSP90B1,NOTCH1,SHH,SMAD4,SNAI1,TP53
KAT2B	Transcription regulator	6.63E‐04	COMMD3‐BMI1,PTCH1,RB1,TP53
HR	Transcription regulator	7.23E‐04	AMD1,DLX3,PLCG1,UBR2
HOXA5	Transcription regulator	7.41E‐04	IHH,SHH,TP53
HTT	Transcription regulator	7.60E‐04	ABCA1,AMD1,CDK2,DLX3,EP300,GCLC,GRK2,GSC,HIF1A,HTT,KAT2A,MTOR,MYL1,OTX2,RAB10,TP53
POU2F1	Transcription regulator	8.22E‐04	ATF2,CDX2,FGF4,HNF1B,ISL1,POU2F1
SNAI1	Transcription regulator	8.22E‐04	CDH5,CDK2,CDK4,SNAI1,THBD,ZEB2
MAFK	Transcription regulator	8.59E‐04	GCLC,TXN,TXNRD1
ID2	Transcription regulator	9.88E‐04	CDK4,CDKN2A,EOMES,HIF1A,MAPK7,NOTCH1,SOX4
TP73	Transcription regulator	1.02E‐03	CDK2,FLT1,G6PD,MYCN,NOTCH1,RB1,SNAI1,SPINT1,TBX5,TP53,XRCC1
TAL1	Transcription regulator	1.05E‐03	AFDN,CDH5,CDK6,CDKN2A,GINS1,NOTCH1,PLCG1,SOX4
FGF4	Growth factor	1.13E‐03	DNMT1,FOXA2,SHH
ZBTB7A	Transcription regulator	1.13E‐03	CDK2,CDKN2A,NRAS
STAT5A	Transcription regulator	1.13E‐03	CASP8,CDC7,CDK4,CDK6,EOMES,EZH2,MCL1,SLC34A2,TP53
Rhox4b (includes others)	Transcription regulator	1.19E‐03	CDH5,TAL1
KLF17	Transcription regulator	1.19E‐03	RB1,TP53
ID3	Transcription regulator	1.25E‐03	CDKN2A,EOMES,HIF1A,MAPK7,NOTCH1,SOX4,TP53
SMAD3	Transcription regulator	1.38E‐03	CDH5,CDK4,CDKN2A,MAX,RAC1,SNAI1,TDGF1,ZEB2
ZEB2	Transcription regulator	1.44E‐03	CDKN2A,EHMT2,PLCG1,SNAI1
MITF	Transcription regulator	1.46E‐03	CDK2,CDKN2A,CHAF1A,HIF1A,OTX2,SNAI1,TDG,TP53
GATA1	Transcription regulator	1.54E‐03	CDK2,CDK4,CDK6,CDKN2A,COPS5,DICER1,MYCN,TAL1
SMARCA4	Transcription regulator	1.56E‐03	ABCA1,CDK2,CDKN2A,COMMD3‐BMI1,FOXA2,GCLC,LDB1,MYL1,PTCH1,RAD50,SHH,SS18,TP53,TXNRD1
DLX5	Transcription regulator	1.62E‐03	GSC,HAND1,SHH
FGF9	Growth factor	1.62E‐03	GCLC,PTCH1,SHH
FOXO4	Transcription regulator	1.64E‐03	ACACA,CASP8,CDH5,HIF1A,RBL2
FOXC2	Transcription regulator	1.65E‐03	MESP1,NOTCH1,SNAI1,TP53
RAX	Transcription regulator	1.66E‐03	NOTCH1,OTX2
E4F1	Transcription regulator	1.66E‐03	DLD,SLC25A19
DMTF1	Transcription regulator	1.66E‐03	CDKN2A,TP53
HDAC5	Transcription regulator	1.76E‐03	ARC,CASP8,MAPK7,SLC8A1
AMH	Growth factor	1.81E‐03	ACVR1,CDKN2A,RBL2
VDR	Transcription regulator	1.81E‐03	ACACA,HIRA,PLCG1,RAD50,SLC34A2,THBD
FOXA2	Transcription regulator	1.92E‐03	CDX2,FOXA2,GATA4,HNF1B,ISL1,SHH,SNAI1
RBPJ	Transcription regulator	2.08E‐03	CDKN2A,EZH2,FGF4,GNA12,SOX4,TDGF1
GSC	Transcription regulator	2.20E‐03	SHH,ZEB2
TSG101	Transcription regulator	2.20E‐03	TP53,TSG101
PAX3	Transcription regulator	2.21E‐03	ARC,ATF2,F2RL1,G6PD,RARRES2,SOX4,TP53
NOTCH1	Transcription regulator	2.31E‐03	CDK2,DLD,FLT1,MYCN,NOTCH1,RB1,SNAI1,TP53
E2F6	Transcription regulator	2.40E‐03	CDC7,DDX11,RAD51,RBBP8
GFI1	Transcription regulator	2.46E‐03	ATF2,CASP8,ISL1,RAF1,RB1
BMP2	Growth factor	2.69E‐03	BMPR2,CDK4,DLX3,IHH,NOTCH1,SMAD4,SPINT1
FOXA1	Transcription regulator	2.81E‐03	CDKN2A,CDX2,FOXA2,HNF1B,ISL1,SHH
TWIST1	Transcription regulator	3.00E‐03	CDKN2A,EZH2,SHH,SNAI1,TP53,ZEB2
KLF2	Transcription regulator	3.00E‐03	APLNR,FLT1,GATA4,HIF1A,TBX5,THBD
SOX9	Transcription regulator	3.02E‐03	CDK4,CDX2,COMMD3‐BMI1,IHH
JUN	Transcription regulator	3.12E‐03	CDKN2A,DICER1,DNMT1,FOXA2,GCLC,RASA1,SHH,SLC8A1,TP53,TXN,ZEB2
EOMES	Transcription regulator	3.14E‐03	APLNR,EOMES,FOXA2,GSC,MESP1
ATF4	Transcription regulator	3.29E‐03	ABCA1,CDKN2A,HSP90B1,IHH,MCL1,XRCC1
TGFB3	Growth factor	3.40E‐03	CDKN2A,F2RL1,SMAD2,SNAI1,ZEB2
TCF3	Transcription regulator	3.41E‐03	AFDN,CDH5,CDK6,CDKN2A,HAND1,HSP90B1,MYCN,NOTCH1,PLK4
RYBP	Transcription regulator	3.49E‐03	CDX2,GATA4
MEOX1	Transcription regulator	3.49E‐03	CDKN2A,GATA4
BCL6B	Transcription regulator	3.49E‐03	CASP8,TP53
TBX21	Transcription regulator	3.54E‐03	CDK6,EOMES,TP53,ZEB2
SP3	Transcription regulator	3.59E‐03	ABCA1,DLX3,FLT1,MYCN,PCYT1A,TP53,TXNRD1
YBX1	Transcription regulator	3.73E‐03	CDK6,CDKN2A,SNAI1,TP53
ETS2	Transcription regulator	3.73E‐03	CDKN2A,CDX2,FLT1,RAF1
ING1	Transcription regulator	3.85E‐03	CDKN2A,SHH,TP53
ZNF217	Transcription regulator	4.13E‐03	EOMES,GATA4,MYCN,TDGF1
TWIST2	Transcription regulator	4.17E‐03	MYCN,SNAI1,ZEB2
MAFF	Transcription regulator	4.24E‐03	GCLC,TXNRD1
FOXD1	Transcription regulator	4.24E‐03	ISL1,SHH
RUVBL2	Transcription regulator	4.24E‐03	ATF2,TP53
CDKN2C	Transcription regulator	4.24E‐03	CDKN2A,PTCH1
HNF1B	Transcription regulator	4.41E‐03	ACVR1,FOXA2,IHH,SNAI1,ZEB2
EGR3	Transcription regulator	4.51E‐03	NF1,NOTCH1,PTPN11
NGF	Growth factor	4.59E‐03	CDK2,CDKN2A,HTT,MYCN,RAC1,RBL2,TXN
STAT1	Transcription regulator	4.63E‐03	ABCA1,CASP8,CDK2,HIF1A,ISL1,SHH,SLC8A1,SMAD2,TP53
GATA3	Transcription regulator	4.70E‐03	CDX2,DLX3,EOMES,NOTCH1,RAD50,TAL1,ZEB2
FOSL1	Transcription regulator	4.78E‐03	CDKN2A,GCLC,SNAI1,THBD
HSF2	Transcription regulator	4.87E‐03	HIF1A,PSMC4,TXN
HIF1A	Transcription regulator	4.89E‐03	CDKN2A,FLT1,HIF1A,MCL1,NOTCH1,SHH,SNAI1,TBX5,TP53,TXN
NFE2L2	Transcription regulator	4.97E‐03	ATF7,COPS5,CUL1,G6PD,GCLC,HSP90B1,PSMC3,TP53,TXN,TXNRD1
BMP10	Growth factor	5.06E‐03	BMPR2,TBX20
NRG4	Growth factor	5.06E‐03	ABCA1,ACACA
FOXD3	Transcription regulator	5.06E‐03	EZH2,FOXA2
HSF4	Transcription regulator	5.06E‐03	FGF4,HIF1A
TFAP4	Transcription regulator	5.24E‐03	CDK2,CDKN2A,SNAI1
CARM1	Transcription regulator	5.24E‐03	CDKN2A,CDX2,GCLC
MEN1	Transcription regulator	5.62E‐03	CASP8,CDK4,EZH2
CTCF	Transcription regulator	5.74E‐03	CDKN2A,GATA4,MYCN,TP53
HDAC4	Transcription regulator	5.81E‐03	ARC,CDKN2A,HIF1A,SLC8A1,SMAD4
TLE1	Transcription regulator	5.94E‐03	CDKN2A,MCL1
SOX7	Transcription regulator	6.03E‐03	DLX3,OTX2,SOX4
EP300	Transcription regulator	6.72E‐03	CDK2,CDKN2A,EP300,EPN1,NOTCH1,PARP1,PCYT1A,RAD51,RB1,TP53
CBX2	Transcription regulator	6.89E‐03	CDKN2A,GATA4
VEGFB	Growth factor	6.89E‐03	CASP8,TP53,TXNRD1
NFKBIA	Transcription regulator	7.04E‐03	ATF2,CASP8,CDK2,DAG1,EOMES,HIF1A,RAC1,SHH,SMAD4,TP53
SKIL	Transcription regulator	7.34E‐03	FOXA2,GSC,TLN1
ARNTL	Transcription regulator	7.34E‐03	ACACA,IHH,TP53
ATN1	Transcription regulator	7.49E‐03	GRK2,KAT2A,MAX,SOX11,SOX4
MYOD1	Transcription regulator	7.75E‐03	ACACA,CDK2,CDX2,DAG1,MYL1,RB1
TBX3	Transcription regulator	7.90E‐03	CDKN2A,TP53
ACTN4	Transcription regulator	7.90E‐03	MYCN,SNAI1
KDM5B	Transcription regulator	7.96E‐03	COMMD3‐BMI1,GATA4,ISL1,SS18,TAL1
SREBF1	Transcription regulator	8.01E‐03	ABCA1,ACACA,CDK4,G6PD,MYL1,PCYT1A,TP53
MYBL2	Transcription regulator	8.31E‐03	CDK2,CDKN2A,FGF4
ANGPT2	Growth factor	8.55E‐03	GATA4,HIF1A,RDH10,SNAI1,TP53,XRCC1
MTA1	Transcription regulator	8.81E‐03	CDKN2A,EHMT2,SNAI1
NDN	Transcription regulator	8.98E‐03	CDKN2A,RBL2
GFM1	Translation regulator	9.04E‐03	ARC
EEF1E1	Translation regulator	9.04E‐03	TP53
PSMD9	Transcription regulator	9.04E‐03	SMAD2
CITED4	Transcription regulator	9.04E‐03	HIF1A
ZBTB48	Transcription regulator	9.04E‐03	CDKN2A
GTF2E1	Transcription regulator	9.04E‐03	TBP
DAZL	Translation regulator	9.04E‐03	CDK2
SUPT4H1	Transcription regulator	9.04E‐03	HTT
CAND1	Transcription regulator	9.04E‐03	CUL1
GTF2F1	Transcription regulator	9.04E‐03	TBP
HOXD12	Transcription regulator	9.04E‐03	SHH
RPS27L	Translation regulator	9.04E‐03	TP53
ESX1	Transcription regulator	9.04E‐03	KRAS
GPS2	Transcription regulator	9.34E‐03	ABCA1,CDK6,SNX1
ERG	Transcription regulator	9.39E‐03	EZH2,FLT1,MYCN,POU2F1,PTPN11,SOX4
FGF8	Growth factor	9.76E‐03	FGF4,LDB1,OTX2,SHH
FOSL2	Transcription regulator	9.88E‐03	ABCA1,SOX4,TP53
CTBP2	Transcription regulator	1.01E‐02	CDH5,EOMES
DAXX	Transcription regulator	1.01E‐02	CASP8,SMAD4
HDAC3	Transcription regulator	1.05E‐02	BECN1,CDKN2A,G6PD,TBX5
ZEB1	Transcription regulator	1.10E‐02	CDKN2A,COMMD3‐BMI1,RBL2
NOG	Growth factor	1.10E‐02	ISL1,PTCH1,SHH
YY2	Transcription regulator	1.13E‐02	TDGF1,TP53
MSTN	Growth factor	1.16E‐02	CDK2,HIF1A,MTOR
BMP6	Growth factor	1.17E‐02	MYCN,SNAI1,TXNRD1,VCL
FMR1	Translation regulator	1.22E‐02	ARC,DAG1,MTOR
COPS5	Transcription regulator	1.26E‐02	HIF1A,TP53
BRD7	Transcription regulator	1.26E‐02	DICER1,RAD51
NRF1	Transcription regulator	1.29E‐02	GCLC,SDHD,TP53
BACH2	Transcription regulator	1.29E‐02	CDKN2A,MCL1,TP53
ZNF202	Transcription regulator	1.39E‐02	ABCA1,CDKN2A
EIF2S1	Translation regulator	1.39E‐02	GCLC,MCL1
NONO	Transcription regulator	1.39E‐02	ACACA,CDKN2A
NAB2	Transcription regulator	1.39E‐02	FLT1,HIF1A
CTGF	Growth factor	1.53E‐02	HIF1A,LIMS1,SOX4,TP53
SCX	Transcription regulator	1.53E‐02	SNAI1,TBX20
MAF	Transcription regulator	1.63E‐02	RAD50,TP53,TXN
SALL4	Transcription regulator	1.67E‐02	COMMD3‐BMI1,FGF4
FOXC1	Transcription regulator	1.67E‐02	MESP1,NOTCH1
DKK1	Growth factor	1.71E‐02	CDKN2A,TP53,TTYH1
NRIP1	Transcription regulator	1.78E‐02	ACACA,CDKN2A,SLC25A19
EIF4B	Translation regulator	1.80E‐02	MCL1
ALX1	Transcription regulator	1.80E‐02	SNAI1
HOXC5	Transcription regulator	1.80E‐02	SHH
ADNP	Transcription regulator	1.80E‐02	TP53
ZBTB49	Transcription regulator	1.80E‐02	RB1
VENTX	Transcription regulator	1.80E‐02	CDKN2A
ZNF326	Transcription regulator	1.80E‐02	RAD50
SNCAIP	Transcription regulator	1.80E‐02	TP53
ZNF197	Transcription regulator	1.80E‐02	HIF1A
BCLAF1	Transcription regulator	1.80E‐02	TP53
ARNTL2	Transcription regulator	1.80E‐02	THBD
PREB	Transcription regulator	1.80E‐02	ABCA1
ONECUT2	Transcription regulator	1.80E‐02	FOXA2
UHRF1	Transcription regulator	1.80E‐02	RB1
DDX20	Transcription regulator	1.80E‐02	TP53
HTATIP2	Transcription regulator	1.82E‐02	SNAI1,TP53
HEY1	Transcription regulator	1.97E‐02	GATA4,TP53
SMARCB1	Transcription regulator	2.02E‐02	CDC7,CDKN2A,MCM10,PLK4,TP53
LEF1	Transcription regulator	2.03E‐02	CDKN2A,PTCH1,TP53
GH1	Growth factor	2.06E‐02	ACVR1,NRAS,SNAI1,XRCC1
FOXL2	Transcription regulator	2.11E‐02	CDKN2A,RSPO3,SOX4
AIRE	Transcription regulator	2.11E‐02	BMP10,EOMES,MGAT1
E2F5	Transcription regulator	2.13E‐02	BECN1,MYCN
NODAL	Growth factor	2.13E‐02	CDK2,FGF4
SOX6	Transcription regulator	2.13E‐02	MYL1,TP53
HEXIM1	Transcription regulator	2.13E‐02	HIF1A,TP53
NFIC	Transcription regulator	2.13E‐02	CDKN2A,TP53
LEP	Growth factor	2.14E‐02	ACACA,ASB4,FLT1,GCLC,ISL1,MCL1,NOTCH1,RAC1,TP53
HOXA9	Transcription regulator	2.21E‐02	CDKN2A,HIRA,RAD51,SHH,SOX4
DTX1	Transcription regulator	2.29E‐02	MCL1,SNAI1
SPDEF	Transcription regulator	2.37E‐02	HIF1A,SMAD2,SMAD4
HOXA10	Transcription regulator	2.46E‐02	FLT1,MAX,MYCN,THBD,TP53
SP4	Transcription regulator	2.64E‐02	ARC,FLT1
NFIL3	Transcription regulator	2.64E‐02	ACACA,EOMES
KLF6	Transcription regulator	2.66E‐02	MCL1,PTCH1,SHH
THAP11	Transcription regulator	2.69E‐02	GATA4
SOX12	Transcription regulator	2.69E‐02	FGF4
TAF3	Transcription regulator	2.69E‐02	GATA4
OSR2	Transcription regulator	2.69E‐02	OSR1
LHX8	Transcription regulator	2.69E‐02	ISL1
GTF2F2	Transcription regulator	2.69E‐02	TBP
SERTAD1	Transcription regulator	2.69E‐02	CDK4
GTF2H1	Transcription regulator	2.69E‐02	TBP
ARID1B	Transcription regulator	2.69E‐02	ARC
HOXB2	Transcription regulator	2.69E‐02	OTX2
RPS14	Translation regulator	2.69E‐02	TP53
L3MBTL2	Transcription regulator	2.69E‐02	CDC7
SOX21	Transcription regulator	2.69E‐02	CDX2
ATF7IP	Transcription regulator	2.69E‐02	CDKN2A
VEZF1	Transcription regulator	2.69E‐02	UBTF
CASP8AP2	Transcription regulator	2.69E‐02	MCL1
MXD3	Transcription regulator	2.69E‐02	MYCN
FOS	Transcription regulator	2.75E‐02	DNMT1,GATA4,HSP90B1,Macf1,Nrg1,SNAI1,SUMO2,TAL1,TP53,TXN
MYB	Transcription regulator	2.98E‐02	NOTCH1,NRAS,SHH,SNAI1
TCF7L1	Transcription regulator	3.00E‐02	EOMES,FOXA2
SOX10	Transcription regulator	3.00E‐02	DAG1,NOTCH1
MLXIPL	Transcription regulator	3.00E‐02	ACACA,HIF1A
NEUROG3	Transcription regulator	3.06E‐02	ACACA,ISL1,XRCC1
TFAP2C	Transcription regulator	3.06E‐02	CDX2,EOMES,RASA1
IFI16	Transcription regulator	3.16E‐02	CDKN2A,GATA4,XRCC1
TBX2	Transcription regulator	3.16E‐02	CDKN2A,DNMT1,EZH2
CREB1	Transcription regulator	3.39E‐02	ABCA1,ACVR2A,ARC,FLT1,MCL1,NF1,NOTCH1,PNO1,RB1,TXN
FGF16	Growth factor	3.57E‐02	SNAI1
PDLIM1	Transcription regulator	3.57E‐02	SNAI1
JDP2	Transcription regulator	3.57E‐02	TP53
HEY2	Transcription regulator	3.57E‐02	GATA4,TBX5
HLX	Transcription regulator	3.57E‐02	CDKN2A,RB1
SUFU	Transcription regulator	3.57E‐02	PTCH1
EAF2	Transcription regulator	3.57E‐02	HIF1A
HOXC13	Transcription regulator	3.57E‐02	CDKN2A
AES	Transcription regulator	3.57E‐02	FOXA2
FOXH1	Transcription regulator	3.57E‐02	FOXA2
TBPL1	Transcription regulator	3.57E‐02	NF1
RAD21	Transcription regulator	3.57E‐02	BMPR2,SOX4
NFYB	Transcription regulator	3.59E‐02	AMD1,CDKN2A,GNA12,RAD51,SNAI1,UHRF1
MRTFA	Transcription regulator	3.60E‐02	CDKN2A,RAC1,TAL1,VCL
CDX2	Transcription regulator	3.68E‐02	CDX2,HNF1B,MYCN,SNAI1
USF1	Transcription regulator	3.72E‐02	ABCA1,CDK4,TP53
IRF8	Transcription regulator	3.76E‐02	ACVRL1,ATF7,NF1,TP53
SOX17	Transcription regulator	3.78E‐02	FOXA2,GATA4
ETV4	Transcription regulator	3.78E‐02	CDKN2A,SHH
EPAS1	Transcription regulator	3.82E‐02	ACACA,FLT1,FXN,HIF1A,NOTCH1
TBX5	Transcription regulator	3.83E‐02	CDK2,GATA4,SLC8A1
PIAS1	Transcription regulator	3.98E‐02	ACACA,MCL1
NFYA	Transcription regulator	4.07E‐02	COMMD3‐BMI1,NOTCH1,POU2F1
YY1	Transcription regulator	4.17E‐02	CDKN2A,DLX3,MYL1,RAD51,TP53,UHRF1
GTF2B	Transcription regulator	4.19E‐02	RBBP8,TBP
XBP1	Transcription regulator	4.24E‐02	ACACA,BECN1,HSP90B1,PCYT1A,TXN
ONECUT1	Transcription regulator	4.35E‐02	ACVR1,CDK2,FOXA2,HNF1B,KAT7,TSG101
MTDH	Transcription regulator	4.40E‐02	CASP8,HIF1A
PGF	Growth factor	4.40E‐02	FLT1,HIF1A
NFE2L1	Transcription regulator	4.40E‐02	GCLC,PSMC3
PRRX2	Transcription regulator	4.44E‐02	SHH
TRIM29	Transcription regulator	4.44E‐02	SNAI1
SSBP2	Transcription regulator	4.44E‐02	LDB1
MTF2	Transcription regulator	4.44E‐02	CDKN2A
RREB1	Transcription regulator	4.44E‐02	CDKN2A
PHF6	Transcription regulator	4.44E‐02	UBTF
TAF9	Transcription regulator	4.44E‐02	TP53
ELAVL4	Translation regulator	4.44E‐02	MYCN
BRCA2	Transcription regulator	4.44E‐02	TP53
BRF1	Transcription regulator	4.44E‐02	TBP
GTF2H4	Transcription regulator	4.44E‐02	CDK4
MYCL	Transcription regulator	4.44E‐02	CDK4
Foxp2	Transcription regulator	4.44E‐02	MYCN
Meis1	Transcription regulator	4.44E‐02	HIF1A
MAX	Transcription regulator	4.57E‐02	CDK4,EZH2,RBBP8
POU4F2	Transcription regulator	4.62E‐02	OTX2,SHH
MYOCD	Transcription regulator	4.70E‐02	GATA4,HAND1,TP53
ATF2	Transcription regulator	4.96E‐02	CDKN2A,MCL1,NOTCH1

**FIGURE 14:**
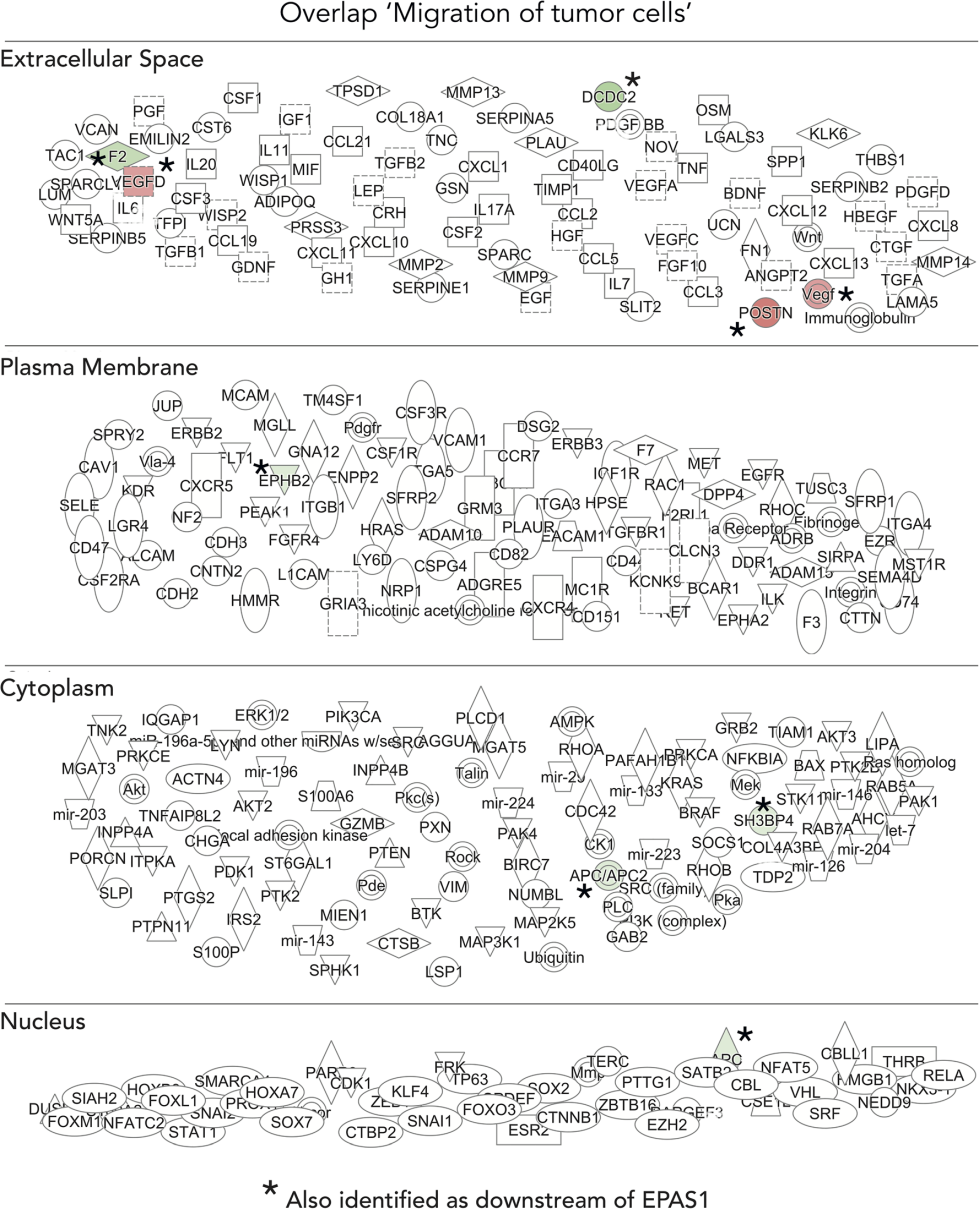
Gene set enrichment analysis identifies key molecules for migration of tumor cells. Deeper analysis of overlap of genes involved in downstream process “migration of tumor cells” (Figure 11A,B) and genes from RNA sequencing data. The shape of molecules and their meaning, that is, correspondence to protein family and so forth, is found here: http://qiagen.force.com/KnowledgeBase/KnowledgeIPAPage?id=kA41i000000L5rTCAS. As an example, the diamond‐shaped molecules correspond to enzymes, oval standing shapes should be read as transmembrane receptors and lying oval shapes are transcription regulators. Green nodes indicate downregulated molecules. The intensity of the color reveals the strength of the expression, that is, the stronger the color the more significant. The dashed lines indicate an indirect interaction between molecules in the network whereas solid lines are direct interactions. The solid arrow explains the direction of the indicated interaction. A line, solid, or dashed, without an arrowhead indicate an RNA‐RNA interaction

Dividing the hits from RNA sequencing data that overlap with genes enriched for migration of tumor cells revealed a large subset of genes that encode plasma membrane associated‐ or are secreted proteins (Figure 14). Several of these overlapping genes were among the 97 significantly differentially expressed (with cut‐off *P* < .005), suggesting a close regulatory relationship between HIF‐2α and migration, potentially mediated by secreted extrinsic cues and/or intercellular signaling, at least during these time points of development.

### 2.13 CDX2 and HNF1B are predicted mediators of observed in vivo phenotypes

**FIGURE 15:**
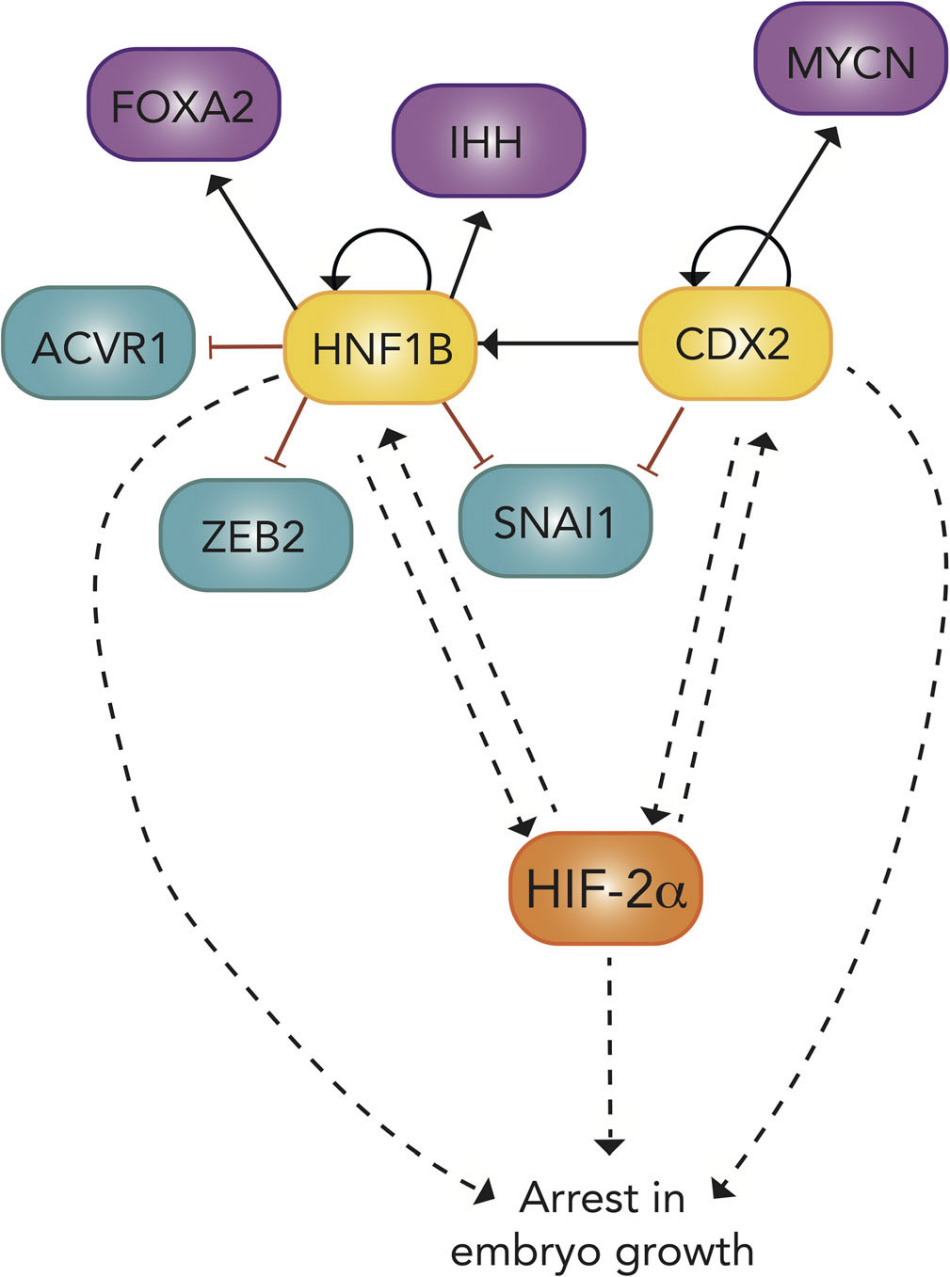
Schematic of the gene regulatory network including *EPAS1* and downstream *CDX2* and *HNF1B* coupled to arrested embryo growth

Two other predicted genes upstream of arrested embryo growth were *CDX2* and *HNF1B*, which were among the 97 significantly differentially expressed genes (DEGs) in the RNA sequencing data (Figures 10C and 13). Deeper analysis of these genes revealed autocrine signaling as well as an interconnected regulation between the two (Figure 15). EMT‐related genes *ZEB2* and *SNAI1* were negatively regulated by both of these genes (Figure 15). In addition, *CDX2* was predicted to regulate *MYCN*, a transcription factor commonly amplified in aggressive neuroblastoma (Figure 15). Both *CDX2* and *HNF1B* were predicted to be upstream regulators of HIF‐2α, as well as downstream targets. The majority of predicted *EPAS1* upstream regulators were indeed transcription factors, and we observed an enrichment for stem cell associated genes, which is in conjunction with previous reports on relationships between HIF‐2α and, for example, OCT4 and NANOG^29^ (Table 5).

**TABLE 5:** Full list of genes identified as potential upstream regulators of HIF‐2α from RNA sequencing data. Target molecules are among the 97 significantly (*P* < .005) DEGs between 5′‐mispair and EPAS1 morpholino samples identified by RNA sequencing

Upstream of EPAS1					
Upstream regulator	Expr log ratio	Molecule type	Activation z‐score	*P*‐value of overlap	Target molecules in dataset
GATA4		Transcription regulator	−0.365	9.39E‐06	CDX2,CLDN1,DLX3,FAP,FSTL4,POSTN,TNNC1
TAF4		Transcription regulator		3.14E‐05	CDH1,CLDN1,DCN,PDGFC,VEGFD
CDX2	−2.439	Transcription regulator		2.26E‐04	APC,CDH1,CDX2,CLDN1,HNF1B
FOXA2		Transcription regulator		1.04E‐03	ALB,CDH1,CDX2,HADH,HNF1B
BMP4		Growth factor		1.17E‐03	CDH1,CDX2,DLX3,POSTN,SCARB1
TBX5		Transcription regulator		2.32E‐03	FAP,POSTN,TNNC1
FOXA1		Transcription regulator		3.33E‐03	CDH1,CDX2,HADH,HNF1B
KLF4		Transcription regulator	−1.324	3.56E‐03	ALB,CDH1,CDX2,DLX3,LIN28A
SKI		Transcription regulator		3.74E‐03	CDH1,FAP
SNAI3		Transcription regulator		3.97E‐03	CDH1
TGFB1		Growth factor	1.679	4.28E‐03	ALB,CDH1,CHST8,CRHR2,DCN,EPHB2,F2,FAP,GLCE,GRHL2,HADH,PDGFC,POSTN,SCARB1,TLE4
KMT2D		Transcription regulator	0.254	5.14E‐03	ASL,SCARB1,TNNC1,WDR17
STAT5A		Transcription regulator		5.44E‐03	CDH1,SNTB1,TLE4,TNNC1,TSPAN8
TFE3		Transcription regulator		5.91E‐03	ALB,CDH1
HOXA7		Transcription regulator		5.91E‐03	CDH1,LY86
CITED2		Transcription regulator		6.74E‐03	CDX2,VEGFD
MYOD1		Transcription regulator	−1.192	6.99E‐03	CDX2,HADH,POSTN,TNNC1
GATA6		Transcription regulator	−0.068	7.38E‐03	CDX2,DLX3,HNF1B,TNNC1
CERS3		Transcription regulator		7.93E‐03	ASAH1
ALX1		Transcription regulator		7.93E‐03	CDH1
LMCD1		Transcription regulator		7.93E‐03	TNNC1
VAX2		Transcription regulator		7.93E‐03	EPHB2
TEAD4		Transcription regulator		8.53E‐03	CDX2,TNNC1
TWIST2		Transcription regulator		9.01E‐03	CDH1,POSTN
BMP2		Growth factor		9.08E‐03	CDH1,DLX3,PLXNA2,POSTN
CEBPA		Transcription regulator	−1.452	9.09E‐03	ALB,ASL,CDH1,DLX3,HNF1B,LIN28A
SOX2		Transcription regulator	−0.529	1.02E‐02	ALB,APC,CDX2,DLX3,LIN28A
TFAP4		Transcription regulator		1.05E‐02	CDH1,CLDN1
POU5F1		Transcription regulator	1.332	1.08E‐02	ALB,CDH1,CDX2,DLX3,LIN28A
CBX5		Transcription regulator		1.14E‐02	SNTB1,TLE4,TSPAN8
HNF1B	−2.282	Transcription regulator		1.17E‐02	ALB,CDH1,CLDN1
ISX		Transcription regulator		1.19E‐02	SCARB1
CERS4		Transcription regulator		1.19E‐02	ASAH1
Tardbp		Transcription regulator		1.19E‐02	ERBB4
EEF1D		Transcription regulator		1.19E‐02	CDH1
SOX21		Transcription regulator		1.19E‐02	CDX2
JMY		Transcription regulator		1.19E‐02	CDH1
POU2F1		Transcription regulator		1.25E‐02	CDX2,HNF1B,TNNC1
MEF2C		Transcription regulator		1.25E‐02	FAP,POSTN,TNNC1
FGF1		Growth factor		1.36E‐02	ALB,CDH1,POSTN
FGF16		Growth factor		1.58E‐02	CDH1
PDLIM1		Transcription regulator		1.58E‐02	CDH1
HAND2		Transcription regulator		1.63E‐02	FAP,TNNC1
VHL		Transcription regulator		1.64E‐02	CDH1,GLCE,SEC61G
FGF2		Growth factor	0.932	1.64E‐02	ALB,CDH1,CDX2,DCN,PDGFC
ZEB1		Transcription regulator		1.76E‐02	CDH1,GRHL2
ETV5		Transcription regulator		1.82E‐02	CDH1,CLDN1
WISP2		Growth factor		1.82E‐02	CDH1,CLDN1
TP63		Transcription regulator	−1.807	1.89E‐02	APC,CDH1,GRHL2,LIN28A,POSTN
FOXI1		Transcription regulator		1.97E‐02	SLC12A3
RFXANK		Transcription regulator		1.97E‐02	EPHA3
NFYB		Transcription regulator		2.24E‐02	APC,CDH1,DPP10,SCARB1
CERS5		Transcription regulator		2.36E‐02	ASAH1
KLF8		Transcription regulator		2.36E‐02	CDH1
HMG20A		Transcription regulator		2.36E‐02	CDH1
WISP3		Growth factor		2.36E‐02	CDH1
MEIS2		Transcription regulator		2.36E‐02	CDH1
ERF		Transcription regulator		2.36E‐02	CDH1
DKK1		Growth factor		2.38E‐02	EPHB2,TNNC1
SNAI2		Transcription regulator		2.46E‐02	CDH1,CLDN1
YY1		Transcription regulator		2.52E‐02	CDH1,DLX3,SCARB1,TNNC1
MEF2D		Transcription regulator		2.53E‐02	CDH1,TNNC1
CEBPB		Transcription regulator	1.103	2.56E‐02	ALB,DCN,DLX3,KRT15,SCARB1
ZNF100		Transcription regulator		2.75E‐02	POSTN
ZNF85		Transcription regulator		2.75E‐02	POSTN
ZNF254		Transcription regulator		2.75E‐02	POSTN
ZNF431		Transcription regulator		2.75E‐02	POSTN
ZNF43		Transcription regulator		2.75E‐02	POSTN
ZNF429		Transcription regulator		2.75E‐02	POSTN
NKX3‐2		Transcription regulator		2.75E‐02	POSTN
EHMT1		Transcription regulator		2.75E‐02	CDH1
FOXN3		Transcription regulator		2.75E‐02	CFAP36
SOX9		Transcription regulator		2.84E‐02	CDX2,KRT15
SPDEF		Transcription regulator		3.00E‐02	APC,PTPRF
MYC		Transcription regulator	−0.039	3.12E‐02	ALB,CDH1,CDX2,DLX3,F2,FAP,SCARB1,SLC25A21,TLE4
CERS6		Transcription regulator		3.14E‐02	ASAH1
TEAD3		Transcription regulator		3.14E‐02	TNNC1
GRIP1		Transcription regulator		3.14E‐02	FRAS1
ZNF91		Transcription regulator		3.14E‐02	POSTN
LEP		Growth factor	−0.169	3.23E‐02	ASAH1,ASL,CDH1,CRHR2,SCARB1
NFKBID		Transcription regulator		3.52E‐02	CDH1
FGF3		Growth factor		3.52E‐02	CDH1
GLIS1		Transcription regulator		3.52E‐02	LIN28A
TFAP2C		Transcription regulator		3.59E‐02	CDH1,CDX2
PDX1		Transcription regulator		3.86E‐02	ALB,PTPRF,TSPAN8
WWC1		Transcription regulator		3.91E‐02	CDH1
PRDM16		Transcription regulator		3.91E‐02	DCN
RYBP		Transcription regulator		3.91E‐02	CDX2
LCOR		Transcription regulator		3.91E‐02	CDH1
INHBA		Growth factor		4.12E‐02	CDH1,CPNE8,ERBB4
CERS2		Transcription regulator		4.29E‐02	ASAH1
RFX2		Transcription regulator		4.29E‐02	DCDC2
GMNN		Transcription regulator		4.32E‐02	CDH1,CDX2
CCND1		Transcription regulator		4.42E‐02	CDH1,JKAMP,MYO7A,RMI2
SMAD2		Transcription regulator		4.60E‐02	CDH1,CDX2
GATA5		Transcription regulator		4.67E‐02	TNNC1
AJUBA		Transcription regulator		4.67E‐02	CDH1
MYOCD		Transcription regulator		4.89E‐02	FAP,TNNC1

Abbreviation: DEGs, differentially expressed genes; HIF, hypoxia inducible factor.

## 3 DISCUSSION

HIF‐2α has been implicated in tumor growth and is expressed in putative cancer stem cells of several tumors including pediatric neuroblastoma, a tumor form likely arising from trunk neural crest. The four published HIF‐2α knockout mice models differ in their resulting phenotype,^17^, ^30^, ^31^, ^32^, ^33^ but one thing they share is that HIF‐2α^−/−^ mice display defects in SNS development—a tissue arising from trunk neural crest. Despite this, little has been known about HIF‐2α expression and function during normal trunk neural crest development following delamination from the neural tube. Here, we show that the HIF‐2α protein is expressed in trunk neural crest cells and sympathetic neuroblasts during normal embryogenesis in three different species, human, mouse and avian and examine its function using the chick embryo as a model amenable to experimental manipulations. Comparable data across human, mouse, and avian tissue suggest that cross‐species interpretation of further results is valid.

HIF‐2α is canonically regulated by oxygen‐dependent prolyl hydroxylases and degraded at high oxygen concentrations. However, it has become clear that mechanisms of regulation and actions of HIF‐2α is much more complex, and that HIF‐2α, for example, can be stabilized at physiological oxygen levels in embryonic and adult as well as tumor tissues. To investigate the oxygen availability in the neural tube at trunk axial level during development, we measured oxygen saturation within the neural tube using a microsensor. Oxygen levels slowly decrease within this tissue over time (HH10‐HH24), with the exception of a temporary rise at HH19. This temporal change is most likely due to relaxed oxygen respiration and a possible temporary reduction in cell proliferation or aerobic respiration that lead to less O_2_ being consumed during this particular developmental stage. We speculate that these changes are coordinated and necessary in the overall developmental program, and that the increase of O_2_ at HH19 therefore highlights that during development both access to oxygen and to molecular regulation of cell behavior—such as that of HIF‐2α, for example,—are involved in the true breed of cells and tissue. Technically impressive papers published in the 1980s measured oxygen concentrations in the organ primordia in the developing chick embryo using microelectrodes, and demonstrated low tissue PO_2_,^34^, ^35^ supporting our observed decrease in oxygen over time. Our previous data on HIF‐2α noncanonical cytoplasmic localization and expression at physiological oxygen levels in tumor tissue,^6^, ^7^, ^9^ together with our results presented here demonstrating dual nuclear and cytoplasmic localization during trunk neural crest development support the need of further research on the complexity of HIF‐2α.

We examined the functional role of HIF‐2α in trunk neural crest development by using overexpression and knockdown approaches. Either knockdown or overexpression of HIF‐2α affected several functions critical for proper embryonic development. Not only do embryos with dysregulated HIF‐2α have developmental delays as compared to their control counterparts, but they also exhibit altered neural crest gene expression profiles. Consistent with observed in vivo effects, RNA sequencing data demonstrate a global genome level change after loss of HIF‐2α, with enrichment of genes involved in invasive behavior and growth arrest. Furthermore, we observed altered trunk neural crest migratory patterns as well as enhanced proliferative capacity of trunk neural crest cells in vivo, as well as in our RNA sequencing data.

Knockdown and overexpression of HIF‐2α confer the same biological phenotypes of trunk neural crest cells in vivo. We hypothesize that this is due to the importance of keeping HIF‐2α levels tightly regulated during development, rather than requiring particularly low or high expression. This is in line with the function and strict regulation of several other transcription factors involved in neural crest cell development and migration.

Despite extensive proliferation of trunk neural crest cells with dysregulated HIF‐2α expression, the embryos as a whole develop at a slower pace than their control counterparts. This could be explained by either, or both, of the following processes. First, several papers have reported that differences in oxygen levels severely affects embryonic growth.^12^, ^13^, ^34^ Considering that physiological HIF‐2α creates a so‐called pseudohypoxic phenotype,^36^ perturbation of HIF‐2α expression levels could be a contributing factor to the decreased growth rate. Secondly, cell division of trunk neural crest cells is in general limited during their active migratory phase. The observed embryonic delay relative to increased trunk neural crest cell proliferation may be the result of a skewed cell division to migration ratio, with increased proliferation possibly causing a failure in timely cell migration.

The capacity to self‐renew is an important feature of stem‐like cells. Our data suggest that *EPAS1* knockout cells exhibit enhanced self‐renewal, in line with observations in neuroblastoma cells with aberrant HIF‐2α expression which are more immature, stem cell‐ and neural crest‐like.^7^ In addition, crestospheres formed by HIF‐2α dysregulated single cells were on average larger, a sign of enhanced proliferative capacity in agreement with our EdU results.

With regard to kinase signaling, RNA sequencing data revealed enrichment of two signaling pathways, the ephrin receptor‐ and PI3K pathways. This suggests that environmental signaling cues may be influencing trunk neural crest behavior. Of note, we have recently identified that PI3K‐mTORC2 regulates HIF‐2α expression and functions as a valid treatment target in neuroblastoma.^27^, ^28^ Genes associated with migration of tumor cells mainly encode for plasma membrane and secreted proteins, including several members of the matrix metalloproteinase (MMP) family. MMPs promote invasion and migration by degrading components of the extracellular matrix and have been shown to be regulated by HIF‐2α in several different tumor forms,^37^, ^38^ further reinforcing a possible connection between HIF‐2α, trunk neural crest cells and invasive migratory behavior.

The stem cell gene *POU5F1*, more commonly known as Oct4, is driven by HIF‐2α in immature cells during development.^29^ We found that Oct4 is predicted to be upstream of arrested embryo growth, but also an upstream regulator of *EPAS1* itself. One of the *EPAS1* target molecules connecting Oct4 and HIF‐2α is *CDX2*, which in turn is upstream of *EPAS1* as well as arrested embryo growth (Figures 13 and 15, and Tables 4 and 5). CDX2 is indeed one of the major players involved in mediating the HIF‐2α driven effects on embryonic development. Considering that CDX2 is an early trunk neural crest marker,^22^ a possible explanation for delayed embryonic development might also be halted trunk neural crest commitment; however, this requires further investigation.

These findings contribute to understanding a complex regulatory network involved in mediating trunk neural crest development. We posit that the cancer associated protein HIF‐2α may play a central role in embryonic growth, global trunk neural crest cell gene expression, migration, proliferation, and self‐renewal features within this network. These findings are in line with data from Ko et al showing that HIF‐2α protects neural progenitor cell survival and differentiation in zebrafish CNS development.^39^ In conclusion, our results highlight the importance of strict control of HIF‐2α levels for maintenance of normal embryonic growth and trunk neural crest development.

## 4 EXPERIMENTAL PROCEDURES

### 4.1 Chick embryos

According to Swedish regulations (Jordbruksverkets föreskrift L150, §5) work on chick embryos younger than embryonic day 13 do not require Institutional Animal Care and Use Committee oversight.

### 4.2 Human and mouse fetal tissue

Human fetal tissue (ethical approval Dnr 6.1.8‐2887/2017, Lund University, Sweden) was obtained from elective abortions. Tissue samples were dissected in custom‐made hibernation medium (Life Technologies, Carlsbad, California) and fixed in 4% formaldehyde overnight. Following a sucrose gradient, embryos were embedded in gelatin for transverse sectioning at 12 μm (ew5) or 7 μm (ew6) using a cryostat.

### 4.3 Cell culture

The human neuroblastoma cell line SK‐N‐BE(2)c (ATCC; Manassas, Virginia) was cultured in MEM supplemented with 10% fetal bovine serum and 100 units penicillin and 10 μg/mL streptomycin. As part of our laboratory routines, all cells were maintained in culture for no more than 30 continuous passages and regularly screened for mycoplasma. SK‐N‐BE(2)c cells were authenticated by SNP profiling (Multiplexion, Germany).

### 4.4 Embryos and perturbations

Chick embryos were acquired from commercially purchased fertilized eggs and incubated at 37.5°C until desired developmental Hamburger Hamilton (HH) stages were reached.^10^ Optimal conditions for high transfection efficiency applying one‐sided electroporation in ovo were determined to 5 pulses of 30 ms each at 22 V. Ringer's balanced salt solution (solution‐1:144 g NaCl, 4.5 g CaCl•2H_2_O, 7.4 g KCl, ddH_2_O to 500 mL; solution‐2:4.35 g Na_2_HPO_4_•7H_2_O, 0.4 g KH_2_PO_4_, ddH_2_O to 500 mL [adjust final pH to 7.4]) containing 1% penicillin/streptomycin was used in all experiments. Morpholinos used were from GeneTools with the following sequences; splice targeting EPAS1 oligo (5′‐GAAAGTGTGAGGGAACAAGTTACCT‐3′) and a corresponding 5′‐mispair oligo (5′‐GAtAcTGTcAGGcAACAAcTTACCT‐3′). Morpholinos were injected at a concentration of 1 mM and co‐electroporated with a GFP tagged empty control vector (1 μg/μL). RFP‐tagged *EPAS1* overexpression construct or corresponding empty control vector were electroporated at a concentration of 2.5 μg/μL. CRISPR constructs with gRNA nontargeting control (#99140, Addgene) or gRNAs targeting *EPAS1* (EPAS1.1.gRNA Top oligo—5′ ggatgGCTCAGAACTGCTCctacc 3′, Bot oligo—5′ aaacggtagGAGCAGTTCTGAGCc 3′; EPAS1.2.gRNA Top oligo—5′ ggatgAAGGCATCCATAATGCGCC 3′, Bot oligo—5′ aaacGGCGCATTATGGATGCCTTc; 3′; EPAS1.3.gRNA Top oligo—5′ ggatgAAATACATGGGTCTCACCC 3′, Bot oligo—5′ aaacGGGTGAGACCCATGTATTTc 3′) were cloned into U6.3 > gRNA.f + e (#99139, Addgene) and electroporated at a concentration of 1.5 μg/μL, and accompanying Cas9‐GFP (#99138, Addgene) at 2 μg/μL.^40^ All constructs were injected at HH stage 10+/11 into the lumen of the neural tube from the posterior end and embryos were electroporated in ovo applying electrodes 4 mm apart, covering the whole embryo. One‐sided electroporation was performed to allow for an internal control side within each individual embryo. Embryos were allowed to sit at room temperature for 6 to 10 hours before further incubation of the embryos at 37.5°C in order to allow the Cas9 protein to fold. Importantly, apart for analysis on embryo growth (ie, age determination), all analyses were performed on sections/cells at the trunk axial level of the embryo.

For harvesting of tissue for RNA extraction, embryos were incubated at 37.5°C for 24 (morpholinos and overexpression vectors) or 36 (CRISPR/Cas9) hours postelectroporation. The trunk portion of neural tubes was dissected and immediately snap frozen before RNA extraction and qPCR analysis.

### 4.5 Cloning

To overexpress HIF‐2α, the *Gallus gallus EPAS1* coding sequence was amplified using the following primers; Fwd:

5′AAACTCGAGGCCACCATGGACTACAAAGACGATGACGACAAGGCAGGTATGACAGCTGACAAGGAGAAG‐3′, Rev 5′‐AAAGCTAGCTCAGGTTGCCTGGTCCAG‐3′ and cloned into the pCI H2B‐RFP vector (Addgene plasmid #92398). For CRISPR/Cas9 targeting, oligos designed to target *EPAS1* at three different locations (EPAS1.1, EPAS1.2, and EPAS1.3) were annealed pairwise at a concentration of 100 μM per oligo using T4 DNA Ligase Buffer in dH_2_O by heating to 95°C for 5 minutes. The annealed oligo reactions were cooled to room temperature and diluted. The U6.3 > gRNA.f + e (#99139, Addgene) vector was digested over night with BsaI‐HF enzyme (New England Biolabs) and gel extracted. gRNAs were cloned into the digested U6.3 > gRNA.f + e vector using T4 DNA Ligase (New England Biolabs) at room temperature for 20 minutes. Successful inserts were identified by colony PCR using U6 sequencing primer and gRNA reverse oligo specific to each *EPAS1* gRNA.

### 4.6 Neural tube dissections for crestosphere cultures

Neural tubes from respective axial levels were carefully dissected out from embryos at designated somite stages. For cranial‐derived cultures, the very anterior tip was excluded, and the neural tube was dissected until the first somite level as previously described.^26^ For trunk‐derived cultures, the neural tube was dissected between somite 10 to 15 as previously described.^24^, ^25^ Pools of neural tubes from four to six embryos were used for each culture.

### 4.7 Crestosphere cell culture

Neural tube derived cells were cultured in NC medium (DMEM with 4.5 g/L glucose (Corning), 7.5% chick embryo extract (MP Biomedicals, Santa Ana,California), 1X B27 (Life Technologies), basic fibroblast growth factor (bFGF, 20 ng/mL) (Peprotech, Stockholm, Sweden), insulin growth factor‐I (IGF‐I, 20 ng/mL) (Sigma Aldrich, Darmstadt, Germany), retinoic acid (RA; 60 nM for cranial and 180 nM for trunk, respectively) (Sigma Aldrich), and 25 ng/mL BMP‐4 (for trunk) (Peprotech)) in low‐adherence T25 tissue culture flasks as described previously.^24^, ^25^

### 4.8 Self‐renewal assay

Chick embryos at developmental HH stage 10+/11 were injected and electroporated with CRISPR/Cas9 constructs and allowed to develop at 37.5°C to reach HH stage 13^+^/14^−^. Crestosphere cultures were established from embryos electroporated with control, EPAS1.1 or EPAS1.2 constructs. Crestospheres were dissociated into single cells using Accutase (Sigma Aldrich; incubation at 37°C for 40 minutes with 1 minute of pipetting every 10 minutes), and individual cells were manually picked using a p10 pipette tip under a microscope. Single cells were transferred to 96‐well plates prepared with 100 μL of NC medium supplemented with RA and BMP‐4.^25^ The absolute number of spheres formed in each well was quantified manually under the microscope. Sphere diameter was manually measured using the ImageJ software (spheres measured n = 33 and n = 27 for CTRL and EPAS1.2, respectively).

### 4.9 EdU pulse chase labeling

Proliferation was measured using the Click‐iT EdU Cell Proliferation kit (Invitrogen #C10337) according to the manufacturer's recommendations with optimizations from Warren et al.^23^ Chick embryos at developmental HH stage 10+/11 were injected and electroporated with morpholino or overexpression constructs and allowed to develop for an additional 24 hours at 37.5°C. Eggs were then reopened and EdU solution (500 μM in PBS‐DEPC) was added. Eggs were resealed and incubated at 37.5°C for another 4 hours before dissection in Ringer's solution and fixed in 4% paraformaldehyde overnight. Embryos were washed in PBS‐DEPC, H_2_O, and 3% BSA in PBS‐DEPC before permeabilization in 0.5% Triton‐X. Embryos were hybridized in reaction cocktail (Click‐iT Reaction buffer, CuSO_4_, Alexa Fluor 488 Azide and reaction buffer additive), washed and DAPI stained. Embryos were after another round of washing processed through a sucrose gradient and embedded in gelatin.

### 4.10 Whole mount in situ hybridization

For whole mount in situ hybridization, embryos were fixed in 4% PFA and washed in DEPC‐PBT. Samples were gradually dehydrated by bringing them to 100% MeOH and kept at −20°C until use. In situ hybridization was performed as previously described.^41^ Embryos were rehydrated back to 100% PBT, treated with Proteinase K/PBT, washed in 2 mg/mL glycine/PBT and postfixed in 4% paraformaldehyde/0.2% glutaraldehyde for 20 minutes. Embryos were then prehybridized in hybridization buffer for 2 hours at 70°C and hybridized with Digoxigenin (DIG)‐labeled TFAP2B probe overnight at 70°C. Embryos were washed in wash solutions I and II (50% formamide, 1% sodium dodecyl sulfate [SDS] and 5X SSC [NaCl and Na citrate] or 2X SSC, respectively), and blocked in 10% sheep serum for 2 hours followed by incubation with an anti‐DIG antibody (1:2000) (Roche) in TBST/1% sheep serum overnight at 4°C. On day 3, embryos were washed in TBST throughout the day and overnight. Embryos were washed in alkaline phosphatase buffer (NTMT; 100 mM NaCl, 100 mM Tris‐Cl [pH 9.5], 50 mM MgCl2, 1%Tween‐20) before visualizing the signal using NBT/BCIP (Sigma Aldrich). Stained embryos were rinsed in PBT for 20 minutes and postfixed in 4% PFA/ 0.1% glutaraldehyde overnight when considered complete. Embryos were then dehydrated in MeOH to be stored at −20°C. Embryos were later embedded in blocks of gelatin for transverse sectioning at 8 μm using a cryostat. Hybridization probe for avian *TFAP2B* was a kind gift from Dr Felipe Vieceli.

### 4.11 RNA sequencing

Chick embryos of stage HH10+/11 were from the posterior end injected with EPAS1 targeting or corresponding 5′‐mispair morpholinos into the lumen of neural tubes and subsequently electroporated for construct uptake. Following 24 hours of incubation at 37.5°C, embryos were removed from the eggs in Ringer's solution. The neural tube portion at the trunk axial level of individual embryos were carefully dissected, removing surrounding mesodermal tissue, and transferred to Eppendorf tubes (neural tube tissue from one embryo per Eppendorf) that were snap frozen. RNA was extracted from each individual neural tube (five samples per condition [EPAS1 and 5′‐mispair, respectively]) using the RNAqueous Micro Kit (Ambion, #AM1931). Sequencing was performed using NextSeq 500 (Illumina). Alignment of reads was performed using the HISAT2 software and the reference genome was from the Ensemble database (*Gallus gallus* 5.0). Expression counts were performed using the StringTie software and DEG analysis was performed using DESeq2. To obtain a relevant working list out of the 1105 significantly DEGs, we set a cut‐off at *P* < .005 and removed all hits that were NA, ending up with 97 genes. Significance (*P* values) was DESeq2 derived.^42^ RNA sequencing data have been deposited in NCBI's Gene Expression Omnibus^43^ and are accessible through GEO Series accession number GSE140319.

### 4.12 Bioinformatics

GSEA for gene ontology, network and functional analyses were generated through the use of Panther database (analyses performed autumn 2018; (http://pantherdb.org/)^44^ together with the Ingenuity Pathway Analysis (IPA) software^45^ (QIAGEN Inc., https://www.qiagenbioinformatics.com/products/ingenuity‐pathway‐analysis). For a hypothesis‐free/exploratory analysis of the 97 DEGs, IPA was used (*P*‐value calculations using right‐tailed Fisher Exact Test). IPA was mainly used for deeper exploration of the data where the biological hypotheses generated for the project were further explored. Here, a hypotheses‐driven approach was taken where the following categories found from the IPA analysis of the 97 DEGs were further investigated; “Cellular Movement,” within the “Molecular and Cellular Function” result category, “Embryonic Development,” within the category “Physiological System Development and Function,” and “Tumor Morphology,” within the “Disease and Disorders” category. These three biological networks were further investigated within the data set at hand. The investigation for the possible overlap and connections between these networks in the context of the data were hence explored.

### 4.13 Cryosections

Fixed embryos were incubated in a sucrose gradient (5% sucrose for 10 minutes and 15% sucrose for 10 minutes up to several hours) followed by incubation in 7.5% gelatin over night at 37°C. Gelatin embedded samples were cryosectioned at 7 to 20 μm.

### 4.14 Immunohistochemistry and immunofluorescence

Immunohistochemistry on mouse fetal tissue for HIF‐2α (NB100‐132, Novus Biologicals) and TH (ab112, Abcam) was performed using Autostainer (Dako). Sections were counterstained with hematoxylin. Detection of HIF‐2α by immunofluorescence was performed on sections from the trunk axial level of embryos (avian and human) that had been harvested, fixed as whole embryos in 4% PFA overnight, incubated in 5% sucrose for 10 minutes, 15% sucrose for 4 hours and gelatin overnight. Embryos were then embedded in gelatin and snap frozen. Dry embryo sections were incubated in ice‐cold acetone followed by 0.3% Triton‐X in PBS. After washing in PBS, slides were blocked in DAKO serum‐free ready‐to‐use block (DAKO, #X0909) for 1 hour before incubation with primary antibodies (in DAKO antibody diluent with background reducing components [DAKO, #S3022]) overnight (HIF‐2α, ab199, Abcam; HNK‐1, 3H5, DSHB). Slides were washed in PBS and incubated with rabbit linker (DAKO, #K8019) followed by secondary antibody in 1% BSA/PBS. Detection of HNK1 and SOX9 by immunofluorescence was performed by blocking (10% goat serum and 0.3% Triton‐X in TBST) of embryo sections followed by incubation with primary antibodies (SOX9, ab5535, Millipore) over night at +4°C. Slides were washed and incubated with secondary antibodies and DAPI for nuclear staining for 1 hour at RT before washing and mounting. Fluorescent images were acquired using an Olympus BX63 microscope, DP80 camera, and cellSens Dimension v 1.12 software (Olympus Cooperation). Detailed information on antibodies can be found in Table 6.

**TABLE 6:** Detailed information of antibodies used in this study

	Species	Dilution	Source	Product #
**IF antibodies**				
*Primary antibody*				
HNK1	Mouse	1:5	Hybridoma bank	3H5
HIF‐2α	Rabbit	1:50	Abcam	ab199
SOX9	Rabbit	1:1000	Millipore	ab5535
*Secondary antibody*				
Anti‐mouse Alexa Fluor‐594	Goat	1:1000	Invitrogen	A‐11032
Anti‐rabbit Alexa Fluor‐546	Donkey	1:1000/1:500	Invitrogen	A‐10040
Anti‐mouse Alexa Fluor‐488	Goat	1:1000	Invitrogen	A‐11008
**IHC antibodies**				
*Primary antibody*				
HIF‐2α	Mouse	1:1000	Novus Biologicals	NB100‐132
HIF‐2α	Rabbit	1:4000	Abcam	ab199
TH	Rabbit	1:1600	Abcam	ab112
**In situ antibodies**				
Anti‐dig‐AP	Mouse	1:2000	Roche Diagnostics	11 093 274 910

**Nuclear staining**				
DAPI		1:3000	Dako	D3571
**Western blot antibodies**				
*Primary antibody*				
HIF‐2α	Rabbit	1:200	Abcam	ab199
SDHA	Mouse	1:4000	Abcam	ab14715
*Secondary antibody*				
Anti‐rabbit	Monkey	1:3000	Invitrogen	65‐6120
Anti‐mouse	Sheep	1:5000	Invitrogen	62‐6520

### 4.15 Western blot

Extracted proteins were separated by SDS‐PAGE, transferred to HyBond‐C‐Extra nitrocellulose membranes, blocked, and incubated with primary antibodies (HIF‐2α, ab199, Abcam; SDHA, ab14715, Abcam) at 4°C overnight. The next day, membranes were incubated with HRP‐conjugated antibodies and proteins detected by ECL solution. Detailed information on antibodies can be found in Table 6.

### 4.16 RNA extraction and quantitative real‐time PCR

Total RNA was extracted using the RNAqueous Micro Kit (Ambion, #AM1931). cDNA synthesis using random primers and qRT‐PCR was performed as previously described.^27^ Relative mRNA levels were normalized to expression of two reference genes (*18S*, *28S*) using the comparative Ct method.^46^ Detailed information of primer sequences can be found in Table 7.

**TABLE 7:** List of primer sequences used for qRT‐PCR analyses

Target gene		5′‐3′
*18S* (reference gene)	Fwd	CCATGATTAAGAGGGACGGC
	Rev	TGGCAAATGCTTTCGCTTT
*28S* (reference gene)	Fwd	GGTATGGGCCCGACGCT
	Rev	CCGATGCCGACGCTCAT
*EPAS1*	Fwd	GGCACCAATACCATGACGA
	Rev	CATGTGCGCGTAACTGTCC
*SOX10*	Fwd	AGCCAGCAATTGAGAAGAAGG
	Rev	GAGGTGCGAAGAGTTGTCC
*B3GAT1*	Fwd	TTGTGGAGGTGGTGAGGA
	Rev	GGCTGTAGGTGGGTGTAATG
*TFAP2B*	Fwd	CCCTCCAAAATCCGTTACTT
	Rev	GGGGACAGAGCAGAACACCT
*HOXC9*	Fwd	TAAGCCACGAAAACGAAGAG
	Rev	GAAGGAAAGTCGGCACAGTC
*HOXA2*	Fwd	AGGCAAGTGAAGGTCTGGTT
	Rev	TCGCCGTTCTGGTTCTCC
*NGFR*	Fwd	AGCAGGAGGAGGTGGAGAA
	Rev	CCCGTGTGAAGCAGTCTATG
*HES6*	Fwd	GCTGATGGCTGATTCCAAAG
	Rev	TCGCAGGTGAGGAGAAGGT
*AGPAT4*	Fwd	TGCTGGGCGTTCTAAATGG
	Rev	ACACTCCTGCTCATCTTCTGG
*HES5*	Fwd	GTATGCCTGGTGCCTCAAA
	Rev	GCTTGTGACCTCTGGAAATG
*RASL11B*	Fwd	GCTGGGCTGTGCTTTCTATG
	Rev	GGTGCTGGTGGTCTGTTGTT
*FMN2*	Fwd	CCATCAGCCAGTCAAGAGGA
	Rev	TAAAGCATCGGGAGCCAAAC
*TAGLN3*	Fwd	AGGCAGCATTTCCAGACC
	Rev	ATGGGTTCGTTTCCCTTTG
*NRCAM*	Fwd	TCATTCCGTGTGATTGCTGT
	Rev	AAGGATTTTCATCGGGGTTT
*EGFP*	Fwd	CCGACCACTACCAGCAGAAC
	Rev	TTGGGGTCTTTGCTCAGG

### 4.17 RNAi experiments

SK‐N‐BE(2)c cells were transfected with ON‐TARGETplus Nontargeting Control siRNA #2 (D‐001810‐02‐05), ON‐TARGETplus siRNA Targeting human HIF1Α (J‐004018‐07) or ON‐TARGETplus siRNA Targeting human EPAS1 (J‐004814‐06), all from Dharmacon, using Lipofectamine 2000 or RNAiMAX. Cells were then placed in 21% or 1% oxygen for 48 hours before harvest. SK‐N‐BE(2)c cells were treated with 200 μM 2,2′‐dipyridyl (DIP), an iron chelator that promotes stabilization of HIF‐α at normoxic conditions for 4 hours before harvest and were used as positive control for western blot detection of HIF‐2α.

### 4.18 Oxygen sensing

Oxygen concentrations were measured through the trunk region of developing chick embryos ex ovo within 30 minutes from dissection using microsensors in a flow system of MQ water. We performed trials to confirm that oxygen concentrations are largely stable within the tissue ex ovo over at least 5 hours. Microprofiles were measured in 50 embryos in developmental stages HH10 to HH24. Embryos were removed from the egg using filter paper as described in Mohlin and Kerosuo,^24^ submerged in a plate with constant flow of newly shaken MQ of room temperature, and immediately measured. Oxygen microsensors were constructed and calibrated as described by Revsbech and Andersen,^47^ mounted on a micromanipulator. The microsensor was manually probing the trunk region and data logged every second. Within the microprofile, 10 consecutive data points of the lowest oxygen concentrations were averaged and set as representing the trunk neural tube. A two‐point calibration was performed using the newly shaken MQ (100% oxygen saturation) and by adding sodium dithionite to nonflowing MQ in the plate after measurements (0% oxygen saturation). Salinity of the tissue was determined using a conductivity meter (WTW 3110) and room temperature noted. The tissue is considered a liquid, where full oxygen saturation at 5‰ salinity and 25°C corresponds to 250 μm/L, 160 mmHg, or 21% atmospheric O_2_. Data were averaged for each HH stage including one measurement of the previous and subsequent HH stages. Replicates vary from 3 to 10 biologically independent data points. Data are presented as percent of maximum saturation in the solution of the specific temperature and salinity.

### 4.19 Quantifications

Embryonic development was quantified in two ways; by determining the HH stage of embryos in ovo using head and tail morphology or by counting the number of somites of dissected embryos ex ovo. The number of embryos (n) for each group is denoted in respective figure legend. The fraction of proliferating EdU^+^ cells was determined by quantifying the number of GFP^+^ proliferating cells as well as RFP^+^ construct targeted cells and dividing the number of double positive cells with the number of RFP^+^ only cells. Premigratory and recently delaminated trunk neural crest cells were included (distinguished by the dotted line in figures). Quantification of migration was performed by calculating the area of detected HNK1 using the ImageJ software. The area of HNK1+ on the electroporated side of the embryos was normalized to that of the control side of the same embryo.

### 4.20 Statistical methods and data sets

One‐way analysis of variance or two‐sided student's unpaired *t* test was used for statistical analyses. For downstream analysis on the 97 DEGs where the software IPA was used, the statistical tests considered were *P*‐value calculations using right‐tailed Fisher exact test.

## CONFLICT OF INTERESTS

The authors declare no conflict of interests.

## AUTHOR CONTRIBUTIONS

**Camilla U. Niklasson**: Investigation, writing‐review and editing. **Elina Fredlund**: Investigation, writing‐review and editing. **Emanuela Monni**: Resources, writing‐review and editing. **Jessica M. Lindvall**: Data curation, formal analysis, software, writing‐review and editing. **Zaal Kokaia**: Resources, writing‐review and editing. **Emma U. Hammarlund**: Formal analysis, investigation, methodology, validation, visualization, writing‐review and editing. **Marianne E. Bronner**: Conceptualization, funding acquisition, methodology, supervision, writing‐review and editing. **Sofie Mohlin**: Conceptualization, data curation, formal analysis, funding acquisition, investigation, methodology, project administration, supervision, validation, visualization, writing‐original draft, writing‐review and editing.

## References

[1] Khudyakov J , Bronner‐Fraser M . Comprehensive spatiotemporal analysis of early chick neural crest network genes. Dev Dyn. 2009;238(3):716‐723. 10.1002/dvdy.21881. 19235729 PMC2650819

[2] Ayer‐Le Lievre CS , Le Douarin NM . The early development of cranial sensory ganglia and the potentialities of their component cells studied in quail‐chick chimeras. Dev Biol. 1982;94(2):291‐310. 10.1016/0012-1606(82)90349-9. 7152108

[3] Bittencourt DA , da Costa MC , Calloni GW , Alvarez‐Silva M , Trentin AG . Fibroblast growth factor 2 promotes the self‐renewal of bipotent glial smooth muscle neural crest progenitors. Stem Cells Dev. 2013;22(8):1241‐1251. 10.1089/scd.2012.0585. 23308383

[4] Bronner‐Fraser M , Fraser SE . Cell lineage analysis reveals multipotency of some avian neural crest cells. Nature. 1988;335(6186):161‐164. 10.1038/335161a0. 2457813

[5] Vega‐Lopez GA , Cerrizuela S , Tribulo C , Aybar MJ . Neurocristopathies: new insights 150 years after the neural crest discovery. Dev Biol. 2018;444(suppl 1):S110‐S143. 10.1016/j.ydbio.2018.05.013. 29802835

[6] Holmquist‐Mengelbier L , Fredlund E , Löfstedt T , et al. Recruitment of HIF‐1alpha and HIF‐2alpha to common target genes is differentially regulated in neuroblastoma: HIF‐2alpha promotes an aggressive phenotype. Cancer Cell. 2006;10(5):413‐423. 10.1016/j.ccr.2006.08.026. 17097563

[7] Pietras A , Gisselsson D , Ora I , et al. High levels of HIF‐2alpha highlight an immature neural crest‐like neuroblastoma cell cohort located in a perivascular niche. J Pathol. 2008;214(4):482‐488. 10.1002/path.2304. 18189331

[8] Pietras A , Hansford LM , Johnsson AS , et al. HIF‐2 alpha maintains an undifferentiated state in neural crest‐like human neuroblastoma tumor‐initiating cells. Proc Natl Acad Sci U S A. 2009;106(39):16805‐16810. 10.1073/pnas.0904606106. 19805377 PMC2745331

[9] Persson CU , von Stedingk K , Fredlund E , et al. ARNT‐dependent HIF‐2 transcriptional activity is not sufficient to regulate downstream target genes in neuroblastoma. Exp Cell Res. 2020;388(2):111845 10.1016/j.yexcr.2020.111845. 31945318

[10] Hamburger V , Hamilton HL . A series of normal stages in the development of the chick embryo. J Morphol. 1951;88(1):49‐92. 24539719

[11] Scully D , Keane E , Batt E , Karunakaran P , Higgins DF , Itasaki N . Hypoxia promotes production of neural crest cells in the embryonic head. Development. 2016;143(10):1742‐1752. 10.1242/dev.131912. 27190038

[12] Nanka O , Valasek P , Dvorakova M , Grim M . Experimental hypoxia and embryonic angiogenesis. Dev Dyn. 2006;235(3):723‐733. 10.1002/dvdy.20689. 16444736

[13] Ota K , Nagai H , Sheng G . Expression and hypoxic regulation of hif1alpha and hif2alpha during early blood and endothelial cell differentiation in chick. Gene Expr Patterns. 2007;7(7):761‐766. 10.1016/j.modgep.2007.05.007. 17625986

[14] Barriga EH , Maxwell PH , Reyes AE , Mayor R . The hypoxia factor Hif‐1alpha controls neural crest chemotaxis and epithelial to mesenchymal transition. J Cell Biol. 2013;201(5):759‐776. 10.1083/jcb.201212100. 23712262 PMC3664719

[15] Hamidian A , Vaapil M , von Stedingk K , et al. Promoter‐associated proteins of EPAS1 identified by enChIP‐MS – a putative role of HDX as a negative regulator. Biochem Biophys Res Commun. 2018;499(2):291‐298. 10.1016/j.bbrc.2018.03.150. 29577908

[16] Påhlman S , Lund LR , Jögi A . Differential HIF‐1alpha and HIF‐2alpha expression in mammary epithelial cells during fat pad invasion, lactation, and involution. PLoS One. 2015;10(5):e0125771 10.1371/journal.pone.0125771. 25955753 PMC4425677

[17] Tian H , Hammer RE , Matsumoto AM , Russell DW , McKnight SL . The hypoxia‐responsive transcription factor EPAS1 is essential for catecholamine homeostasis and protection against heart failure during embryonic development. Genes Dev. 1998;12(21):3320‐3324. 10.1101/gad.12.21.3320. 9808618 PMC317225

[18] Bishop T , Gallagher D , Pascual A , et al. Abnormal sympathoadrenal development and systemic hypotension in PHD3−/− mice. Mol Cell Biol. 2008;28(10):3386‐3400. 10.1128/MCB.02041-07. 18332118 PMC2423159

[19] Mohlin S , Hamidian A , Påhlman S . HIF2A and IGF2 expression correlates in human neuroblastoma cells and normal immature sympathetic neuroblasts. Neoplasia. 2013;15(3):328‐334. 10.1593/neo.121706. 23479510 PMC3593155

[20] Li Z , Bao S , Wu Q , et al. Hypoxia‐inducible factors regulate tumorigenic capacity of glioma stem cells. Cancer Cell. 2009;15(6):501‐513. 10.1016/j.ccr.2009.03.018. 19477429 PMC2693960

[21] Murko C , Vieceli FM , Bronner M . Transcriptome dataset of trunk neural crest cells migrating along the ventral pathway of chick embryos. Data Brief. 2018;21:2547‐2553. 10.1016/j.dib.2018.11.109. 30761336 PMC6288396

[22] Frith TJ , Granata I , Wind M , et al. Human axial progenitors generate trunk neural crest cells in vitro. Elife. 2018;10:7 10.7554/eLife.35786. PMC6101942 30095409

[23] Warren M , Puskarczyk K , Chapman SC . Chick embryo proliferation studies using EdU labeling. Dev Dyn. 2009;238(4):944‐949. 10.1002/dvdy.21895. 19253396 PMC2664394

[24] Mohlin S , Kerosuo L . In vitro maintenance of multipotent neural crest stem cells as crestospheres. Methods Mol Biol. 2019;2002:1‐11. 10.1007/7651_2018_180. 30159826 PMC8014252

[25] Mohlin S , Kunttas E , Persson CU , et al. Maintaining multipotent trunk neural crest stem cells as self‐renewing crestospheres. Dev Biol. 2019;447(2):137‐146. 10.1016/j.ydbio.2019.01.010. 30664880 PMC6497816

[26] Kerosuo L , Nie S , Bajpai R , Bronner ME . Crestospheres: long‐term maintenance of multipotent, premigratory neural crest stem cells. Stem Cell Rep. 2015;5(4):499‐507. 10.1016/j.stemcr.2015.08.017. PMC4625028 26441305

[27] Mohlin S , Hamidian A , von Stedingk K , et al. PI3K‐mTORC2 but not PI3K‐mTORC1 regulates transcription of HIF2A/EPAS1 and vascularization in neuroblastoma. Cancer Res. 2015;75(21):4617‐4628. 10.1158/0008-5472.CAN-15-0708. 26432405

[28] Mohlin S , Hansson K , Radke K , et al. Anti‐tumor effects of PIM/PI3K/mTOR triple kinase inhibitor IBL‐302 in neuroblastoma. EMBO Mol Med. 2019;11(8):e10058 10.15252/emmm.201810058. 31310053 PMC6685085

[29] Covello KL , Kehler J , Yu H , et al. HIF‐2alpha regulates Oct‐4: effects of hypoxia on stem cell function, embryonic development, and tumor growth. Genes Dev. 2006;20(5):557‐570. 10.1101/gad.1399906. 16510872 PMC1410808

[30] Patel SA , Simon MC . Biology of hypoxia‐inducible factor‐2alpha in development and disease. Cell Death Differ. 2008;15(4):628‐634. 10.1038/cdd.2008.17. 18259197 PMC2882207

[31] Peng J , Zhang L , Drysdale L , Fong GH . The transcription factor EPAS‐1/hypoxia‐inducible factor 2alpha plays an important role in vascular remodeling. Proc Natl Acad Sci U S A. 2000;97(15):8386‐8391. 10.1073/pnas.140087397. 10880563 PMC26957

[32] Compernolle V , Brusselmans K , Acker T , et al. Loss of HIF‐2alpha and inhibition of VEGF impair fetal lung maturation, whereas treatment with VEGF prevents fatal respiratory distress in premature mice. Nat Med. 2002;8(7):702‐710. 10.1038/nm721. 12053176

[33] Scortegagna M , Ding K , Oktay Y , et al. Multiple organ pathology, metabolic abnormalities and impaired homeostasis of reactive oxygen species in Epas1−/− mice. Nat Genet. 2003;35(4):331‐340. 10.1038/ng1266. 14608355

[34] Meuer HJ , Hartmann V , Jopp S . Tissue PO2 and growth rate in early chick embryos. Respir Physiol. 1992;90(2):227‐237. 10.1016/0034-5687(92)90083-9. 1494722

[35] Meuer HJ , Baumann R . Oxygen supply of early chick embryo in normoxia and hypoxia. J Exp Zool Suppl. 1987;1:203‐207. 3598492

[36] Påhlman S , Mohlin S . Hypoxia and hypoxia‐inducible factors in neuroblastoma. Cell Tissue Res. 2018;372(2):269‐275. 10.1007/s00441-017-2701-1. 29032465 PMC5915502

[37] Petrella BL , Lohi J , Brinckerhoff CE . Identification of membrane type‐1 matrix metalloproteinase as a target of hypoxia‐inducible factor‐2 alpha in von Hippel‐Lindau renal cell carcinoma. Oncogene. 2005;24(6):1043‐1052. 10.1038/sj.onc.1208305. 15592504 PMC1847637

[38] Koh MY , Lemos R Jr , Liu X , Powis G . The hypoxia‐associated factor switches cells from HIF‐1alpha‐ to HIF‐2alpha‐dependent signaling promoting stem cell characteristics, aggressive tumor growth and invasion. Cancer Res. 2011;71(11):4015‐4027. 10.1158/0008-5472.CAN-10-4142. 21512133 PMC3268651

[39] Ko CY , Tsai MY , Tseng WF , et al. Integration of CNS survival and differentiation by HIF2alpha. Cell Death Differ. 2011;18(11):1757‐1770. 10.1038/cdd.2011.44. 21546908 PMC3190110

[40] Gandhi S , Piacentino ML , Vieceli FM , Bronner ME . Optimization of CRISPR/Cas9 genome editing for loss‐of‐function in the early chick embryo. Dev Biol. 2017;432(1):86‐97. 10.1016/j.ydbio.2017.08.036. 29150011 PMC5728388

[41] Acloque H , Wilkinson DG , Nieto MA . In situ hybridization analysis of chick embryos in whole‐mount and tissue sections. Methods Cell Biol. 2008;87:169‐185. 10.1016/S0091-679X(08)00209-4. 18485297

[42] Love MI , Huber W , Anders S . Moderated estimation of fold change and dispersion for RNA‐seq data with DESeq2. Genome Biol. 2014;15(12):550 10.1186/s13059-014-0550-8. 25516281 PMC4302049

[43] Edgar R , Domrachev M , Lash AE . Gene expression omnibus: NCBI gene expression and hybridization array data repository. Nucleic Acids Res. 2002;30(1):207‐210. 10.1093/nar/30.1.207. 11752295 PMC99122

[44] Thomas PD , Campbell MJ , Kejariwal A , et al. PANTHER: a library of protein families and subfamilies indexed by function. Genome Res. 2003;13(9):2129‐2141. 10.1101/gr.772403. 12952881 PMC403709

[45] Kramer A , Green J , Pollard J Jr , Tugendreich S . Causal analysis approaches in ingenuity pathway analysis. Bioinformatics. 2014;30(4):523‐530. 10.1093/bioinformatics/btt703. 24336805 PMC3928520

[46] Vandesompele J , de Preter K , Pattyn F , et al. Accurate normalization of real‐time quantitative RT‐PCR data by geometric averaging of multiple internal control genes. Genome Biol. 2002;3(7):RESEARCH0034 10.1186/gb-2002-3-7-research0034. 12184808 PMC126239

[47] Revsbech P , Andersen G . Diurnal variation in peak expiratory flow rate among grain elevator workers. Br J Ind Med. 1989;46(8):566‐569. 10.1136/oem.46.8.566. 2775676 PMC1009827

